# Null *cyp1b1* Activity in Zebrafish Leads to Variable Craniofacial Defects Associated with Altered Expression of Extracellular Matrix and Lipid Metabolism Genes

**DOI:** 10.3390/ijms22126430

**Published:** 2021-06-16

**Authors:** Susana Alexandre-Moreno, Juan-Manuel Bonet-Fernández, Raquel Atienzar-Aroca, José-Daniel Aroca-Aguilar, Julio Escribano

**Affiliations:** 1 Área de Genética, Facultad de Medicina de Albacete, Instituto de Investigación en Discapacidades Neurológicas (IDINE), Universidad de Castilla-La Mancha, 02006 Albacete, Spain; Susana.Alexandre@uclm.es (S.A.-M.); juanm.bonet@uclm.es (J.-M.B.-F.); raquel.atienzar@uclm.es (R.A.-A.); 2 Cooperative Research Network on Age-Related Ocular Pathology, Visual and Life Quality (OFTARED), Instituto de Salud Carlos III, 28029 Madrid, Spain

**Keywords:** *CYP1B1*, craniofacial development, CRISPR/Cas9, congenital glaucoma, *cyp1b1*-KO zebrafish

## Abstract

**Simple Summary:**

CYP1B1 is a cytochrome P450 monooxygenase involved in oxidative metabolism of different endogenous lipids and drugs. The loss of function (LoF) of this gene underlies many cases of recessive primary congenital glaucoma (PCG), an infrequent disease and a common cause of infantile loss of vision in children. To the best of our knowledge, this is the first study to generate a *cyp1b1* knockout zebrafish model. The zebrafish line did not exhibit glaucoma-related phenotypes; however, adult mutant zebrafish presented variable craniofacial alterations, including uni- or bilateral craniofacial alterations with incomplete penetrance and variable expressivity. Transcriptomic analyses of seven-dpf *cyp1b1*-KO zebrafish revealed differentially expressed genes related to extracellular matrix and cell adhesion, cell growth and proliferation, lipid metabolism and inflammation. Overall, this study provides evidence for the complexity of the phenotypes and molecular pathways associated with *cyp1b1* LoF, as well as for the dysregulation of extracellular matrix gene expression as one of the mechanisms underlying *cyp1b1* disruption-associated pathogenicity.

**Abstract:**

*CYP1B1* loss of function (LoF) is the main known genetic alteration present in recessive primary congenital glaucoma (PCG), an infrequent disease characterized by delayed embryonic development of the ocular iridocorneal angle; however, the underlying molecular mechanisms are poorly understood. To model *CYP1B1* LoF underlying PCG, we developed a *cyp1b1* knockout (KO) zebrafish line using CRISPR/Cas9 genome editing. This line carries the c.535_667del frameshift mutation that results in the 72% mRNA reduction with the residual mRNA predicted to produce an inactive truncated protein (p.(His179Glyfs*6)). Microphthalmia and jaw maldevelopment were observed in 23% of F0 somatic mosaic mutant larvae (144 hpf). These early phenotypes were not detected in *cyp1b1*-KO F3 larvae (144 hpf), but 27% of adult (four months) zebrafish exhibited uni- or bilateral craniofacial alterations, indicating the existence of incomplete penetrance and variable expressivity. These phenotypes increased to 86% in the adult offspring of inbred progenitors with craniofacial defects. No glaucoma-related phenotypes were observed in *cyp1b1* mutants. Transcriptomic analyses of the offspring (seven dpf) of *cyp1b1*-KO progenitors with adult-onset craniofacial defects revealed functionally enriched differentially expressed genes related to extracellular matrix and cell adhesion, cell growth and proliferation, lipid metabolism (retinoids, steroids and fatty acids and oxidation–reduction processes that include several cytochrome P450 genes) and inflammation. In summary, this study shows the complexity of the phenotypes and molecular pathways associated with *cyp1b1* LoF, with species dependency, and provides evidence for the dysregulation of extracellular matrix gene expression as one of the mechanisms underlying the pathogenicity associated with *cyp1b1* disruption.

## 1. Introduction

CYP1B1 is a cytochrome P450 monooxygenase that participates in the oxidative metabolism of different endogenous lipids including steroids [1], arachidonic acid [2] (the primary source of fatty acids) and retinoids [2,3], and it is also involved in drug metabolism [4]. The human *CYP1B1* gene is located on chromosome 2p22-21 and comprises three exons, with the coding region starting in the second exon and ending in the last exon [5]. This gene encodes an approximately 50-kDa transmembrane protein that is anchored to the endoplasmic reticulum membrane and the inner mitochondrial membrane by a transmembrane amino terminus domain [6]. Structurally, the protein consists of several domains such as a hydrophobic amino-terminal region, a proline-rich region (hinge region) and a carboxyl-terminal portion. This last region contains a set of conserved core structures and a substrate-binding region, including an iron protoporphyrin IX (heme) prosthetic group ligated to cysteine thiolate [7].

Loss-of-function (LoF) variants in the human *CYP1B1* gene [8] are the main known genetic cause of autosomal recessive congenital glaucoma (CG) in different populations [9,10,11,12]. Although CG is an infrequent disease, it is the most common glaucoma in the neonatal and infant period and it is also a major cause of visual loss in children [13]. Abnormal development of the embryonic iridocorneal angle underlies CG through poorly understood mechanisms, although CYP1B1 is hypothesized to metabolize a yet unidentified compound required for normal formation of iridocorneal structures [14]. An altered ECM of the TM, a general feature of PCG [15,16,17], is also present in patients carrying null and hypomorphic *CYP1B1* genotypes [18]. In addition to *CYP1B1*, other genes such as *LTBP2* [19,20], *MYOC* [21], *TEK* [22], *FOXC1* [23] and *CPAMD8* [24,25] are involved in a few congenital glaucoma cases. Genes such as *GPATCH3* [26] and *GUCA1C* [27] have been identified as candidate CG genes, although their role in the disease remains to be confirmed. Remarkable phenotypic variability is also present in *CYP1B1*-associated glaucoma, ranging from mild adult-onset goniodysgenesis to agenesis of the Schlemm canal [18,28] and complete aniridia [29]. This phenomenon suggests the existence of modifier factors in the phenotypic outcome. In fact, rare variants of *FOXC2* and *PITX2* associated with mild functional alterations have been identified as possible modifiers in congenital glaucoma [30]. Previously, we reported that approximately 30% of Spanish CG patients carry either homozygous or compound heterozygous *CYP1B1* LoF variants, often resulting in null genotypes [12]. Even among the cases with null CYP1B1 enzymatic activity which can be considered natural human knockouts, remarkable phenotypic variation is present [12,31]. These facts, along with the existence of incomplete penetrance and the discovery of a significant proportion of patients who carry nondominant heterozygous *CYP1B1* mutations [12], support the importance of genetic and/or environmental modifier factors in CG pathogenesis.

The function of *CYP1B1* has been explored in different animal models. *Cyp1b*-KO mice have ocular drainage structure abnormalities resembling those reported in human PCG patients, and in this animal model, tyrosinase gene (*Tyr*) deficiency increases the magnitude of dysgenesis, indicating that *Tyr* is a modifier of the ocular drainage structure phenotype, although no intraocular pressure increase was detected in these animals [32]. Further studies have reported modest elevation of the intraocular pressure in *Cyp1b1*-KO mice [33] and altered distribution of TM collagen [33,34] associated with decreased levels of periostin [33], as well as TM endothelial dysfunction [34]. Oxidative stress [33,35,36,37,38,39], cell adhesion and migration [37,40] and lipid metabolism [41,42,43] are also altered in *Cyp1b1*-KO mice, suggesting a multifunctional role of this gene in development and homeostasis. *Cyp1b1* LoF has been explored in zebrafish mainly by morpholino (MO)-mediated knockdown [44,45,46,47]. This approach, which inhibits protein expression only in early developmental stages, results in heart malformations and pericardial edema and also affects the development of neural crest cell-derived tissues [47], indicating the role of *cyp1b1* in early embryo development. Overexpression of *cyp1b1* leads to craniofacial and ocular defects, inhibited ocular fissure closure via an RA-independent pathway and disruption of ocular neural crest cell migration. Interestingly, these studies support the existence of functional conservation between the human and zebrafish *cyp1b1* genes [45].

To the best of our knowledge, herein we report the first *cyp1b1*-KO zebrafish model for exploring the pathogenic mechanisms involved in *cyp1b1* LoF. We show that *cyp1b1* inactivation does not mimic congenital glaucoma but leads to adult-onset and variable craniofacial alterations. Transcriptomic analysis reveals alteration of genes participating in extracellular matrix (ECM) and cell adhesion, developmental signaling pathways, lipid metabolism and inflammation. The established *cyp1b1*-KO zebrafish line provides a new model with which to investigate the biological function of this gene and opens new avenues for studying the molecular mechanisms underlying *cyp1b1* LoF-associated pathogenesis.

## 2. Results

### 2.1. Generation and Characterization of a Cyp1b1-KO Zebrafish Line

**Figure 1:**
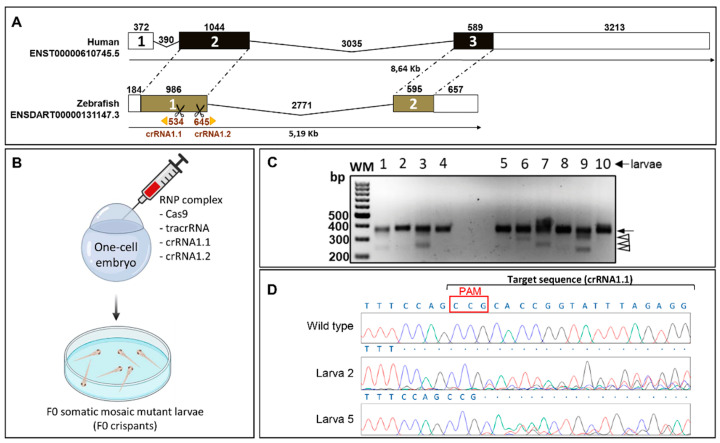
*CYP1B1* gene structure and generation of the zebrafish *cyp1b1* FO by CRISPR/Cas9 genome editing. (**A**) Comparison of exon and intron organization of human and zebrafish *CYP1B1* genes. The numbers above boxes and black lines represent the length in bp of exons and introns, respectively. Untranslated regions are represented by white boxes and coding regions are represented by black and brown boxes. Dotted lines represent conserved exons. The Ensembl region comparison tool was used to obtain the scheme. Scissors indicate the position of the two CRISPR guides used to generate *cyp1b1*-KO (crRNA1.1 and crRNA1.2). (**B**) Obtention of F0 somatic mosaic mutant larvae by the RNP complex (Cas9 protein/tracrRNA/crRNA 1.1/crRNA 1.2) microinjection of one-cell embryos (F0 somatic mosaic crispants, *n* = 215). The two crRNAs were injected simultaneously to generate *cyp1b1* deletions. The scheme was created with the Biorender tool (https://biorender.com/) (accessed on 21 April 2021). (**C**) Analysis of the CRISPR/Cas9 efficiency by agarose electrophoresis of *cyp1b1* exon 1 PCR products amplified from 10 F0 larvae (48 hpf). Black arrow: main PCR product. Arrowheads: different exon 1 deletions. (**D**) Sanger sequencing of the main band (arrow) detected in (**C**). The electropherograms of two larvae (2 and 5) are shown as representative results of this analysis. Overlapping peaks indicate the presence of different mutations. Red box: protospacer adjacent motif (PAM) site.

The overall *CYP1B1* gene structure is conserved between human and zebrafish, although the 5’UTR region is separated from the coding sequence in the human gene (exon 1) and the human 3’UTR is much longer than that of zebrafish (Figure 1A). The coding region of both genes presents a high degree of nucleotide sequence similarity (59%), and the proteins have 57% amino acid identity (Figure S1). To study the effect of both somatic mosaic and germinal *cyp1b1* LoF in zebrafish and to facilitate genotyping by PCR and agarose electrophoresis, we employed CRISPR/Cas9 genome editing simultaneously using two crRNA-targeting nucleotide sequences located 132 bp apart in opposite strands of the coding sequence of exon 1 (Figure 1A, scissors).

**Figure 2:**
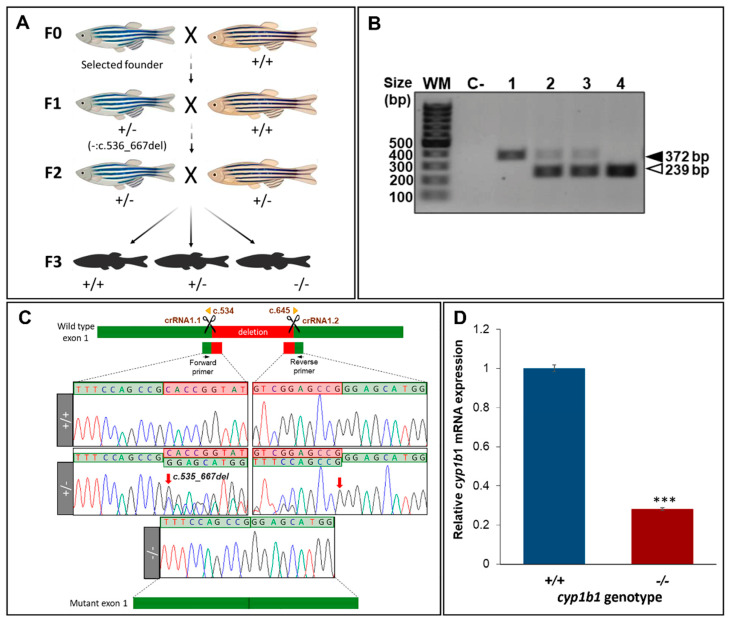
Generation and molecular characterisation of a *cyp1b1*-KO zebrafish line using CRISPR/Cas9 genome editing. (**A**) Stepwise procedure followed to generate the KO line. Adult F0 zebrafish were crossed with wildtype AB animals and the offspring were genotyped by PCR and agarose gel electrophoresis to identify germline transmission of *cyp1b1* deletions (F0 founders). The selected F0 founder was mated with a wildtype AB animal to obtain mutant F1 heterozygotes that were further outcrossed to segregate off-target mutations in the F2 generation. F2 heterozygotes were inbred to obtain F3 fishes. The scheme was created with the Biorender tool (https://biorender.com/) (accessed on 21 April 2021). (**B**). Genotyping by PCR and agarose gel electrophoresis of a 133-bp *cyp1b1 d*eletion in the F3 generation. Representative examples of the three genotypes are shown. Black arrowhead: wildtype allele (372 bp). White arrowhead: mutant allele (239 bp). (**C**) Sanger sequencing of the selected mutation. The top diagram indicates localization of the identified deletion in *cyp1b1* exon 1. Scissors: DNA cleavage sites targeted by the two crRNAs. The numbers correspond to cDNA nucleotide positions. Red arrows in the electropherograms indicate the 5′ and 3′ ends of the deletion. Deleted nucleotides are indicated in the red background. The diagram in the bottom represents the mutant exon. (**D**) Decreased mRNA levels in *cyp1b1*-KO zebrafish. Pools of 45 F4 zebrafish larvae (48 hpf) were used to quantify relative *cyp1b1* mRNA levels by RT-qPCR. The values represent the average of three independent experiments carried out in triplicate. Asterisks indicate statistical significance compared to the wild type, *p* < 0.001 (***).

The RNP complexes (crRNA 1.1/crRNA 1.2/tracrRNA and Cas9 protein) were injected into the animal pole of AB zebrafish at the one-cell stage of development (*n* = 215; Figure 1B). Electrophoretic analysis of the PCR products of *cyp1b1* exon 1 amplified from 10 F0 larvae (48 hpf) revealed a common band of approximately 370 bp (Figure 1C, arrow) and the presence of additional bands ranging from about 250 bp to 350 bp in at least seven embryos, indicating the existence of 50–130-bp deletions (Figure 1C, arrowheads). Sanger sequencing of the purified upper band revealed the presence of multiple peaks downstream of the protospacer adjacent motif site in more than 80% of the embryos (Figure 1D), indicating the presence of different indels originated by Cas9. These results suggest that CRISPR/Cas9 gene editing is highly effective and that most of the injected embryos, including those with deletions not clearly detectable by agarose electrophoresis (Figure 1D, larvae 2 and 5), were somatic mosaics for CRISPR/Cas9-mediated mutations (crispants). Sixty-five F0 crispants were raised to adulthood and screened for the presence of germline-transmitted *cyp1b1* deletions by electrophoretic analysis of exon 1 amplicon as indicated in the Methods section. We selected one F0 founder male zebrafish transmitting a 133-bp deletion (c.535_667del, Figure 2A,B) which was predicted to result in a frameshift and a premature termination codon in the new reading frame (p.(His179Glyfs*6)).

This mutation was also expected to lead to a complete *cyp1b1* LoF by nonsense-mediated mRNA decay (NMD) [48]. The founder male zebrafish was outbred with a wildtype AB female to obtain the F1 generation (Figure 2A). A total of 16 F1 zebrafish were genotyped and eight (50%) were heterozygous for the founder mutation. The F1 heterozygotes were outbred again with wildtype AB zebrafish to further segregate possible off-target mutations (F2 offspring; Figure 2A), and F2 heterozygotes were then inbred to obtain F3 homozygous mutant *cyp1b1* zebrafish (Figure 2A). F3 genotyping by electrophoretic analysis and Sanger sequencing (Figure 2B,C) showed agreement of the proportions of the three genotypes with the expected Mendelian ratios, indicating that *cyp1b1* disruption does not affect zebrafish fertility and viability.

To confirm the proposed NMD degradation of the mutant *cyp1b1* mRNA, we analyzed mRNA levels by RT-qPCR and fluorescent in situ hybridization in the offspring (48 hpf) of inbred F3 homozygotes (F4). RT-qPCR revealed an approximately 70% reduction of *cyp1b1* expression compared to wildtype levels (Figure 2D). In addition, in situ hybridization showed the presence of a *cyp1b1* mRNA signal in the ocular fissure of wildtype embryos as previously described [47], but it was undetectable in the eyes of *cyp1b1*-KO embryos (Figure S2, white arrow). Both results supported the predicted LoF induced by the *cyp1b1* c.535_667del133 mutation via NMD mRNA degradation.

### 2.2. F0 Embryo Development Delay and Variable Craniofacial Defects in Adult Zebrafish due to Cyp1b1 LoF

**Figure 3:**
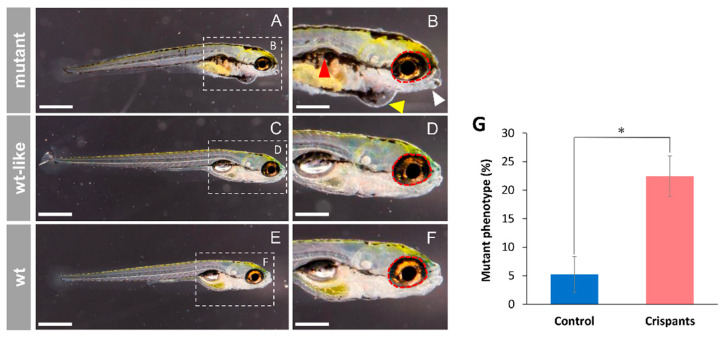
*Cyp1b1* F0 crispants’ phenotypes (144 hpf). (**A**–**D**) One-cell embryos were microinjected with CRISPR/Cas9 ribonucleoprotein complexes targeting *cyp1b1* exon 1. Crispants’ morphology was assessed microscopically at 144 hpf. (**E**,**F**) Non-injected larvae were used as controls (wt). White arrow: lower jaw underdevelopment. Red arrowhead: altered swim bladder development. Red dotted circle: wildtype ocular periphery is indicated as a reference to show microphthalmia. Yellow arrowhead: pericardial edema. Scale bar in (**A**,**C**,**E**) = 500 µm. Scale bar in (**B**,**D**,**F**) = 250 µm. (**G**) Proportion of F0 mutant phenotypes. The controls were microinjected with all CRISPR/Cas9 reagents except crRNAs. Asterisks indicate statistical significance compared to the control, *p* < 0.05 (*). The values correspond to the means ± SEM of three independent experiments (50–80 embryos per group and experiment).

Gross morphological analysis of CRISPR/Cas9-microinjected embryos revealed that at 144 hpf, *cyp1b1* crispants presented a variable combination of morphological alterations consisting of lower jaw underdevelopment, microphthalmia and/or pericardial edema (Figure 3A,B and Figure S3, white arrowhead, red circle and yellow arrowhead, respectively). In addition, some F0 crispants also showed reduced axial length and delayed or absent swim bladder development (Figure 3A,B, red arrowhead). Only 5.2% of the control microinjected embryos showed abnormal morphology at 144 hpf (Figure 3G) compared with 22.5% of the crispants, indicating that the crispants’ phenotypes were specific. Therefore, most F0 crispants presented wild type-like phenotypes similar to those of the non-injected controls (Figure 3C,D,E,F). Comparable results were observed when each crRNA was microinjected individually (Figure S4), indicating that possible off-targets do not influence the observed morphological defects.

**Figure 4:**
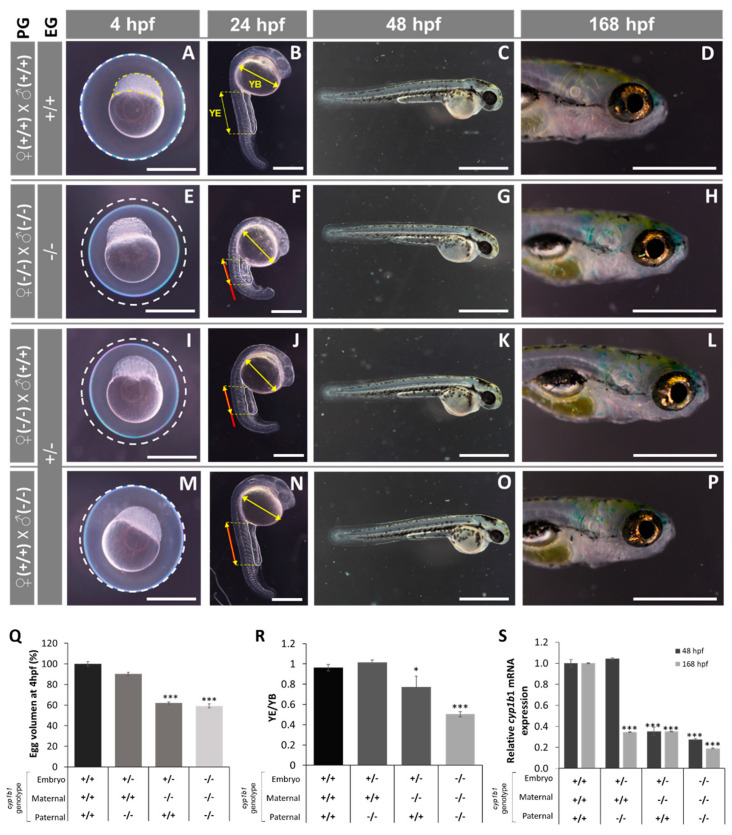
Early (4 -168 hpf) phenotypes of the established *cyp1b1*-KO zebrafish line. (**A**–**P**) The phenotypes were analyzed in F4 zebrafish resulting from inbreeding of young (< six months) F3 siblings. Progenitors’ genotypes (PG) and embryos’ genotypes (EG) are indicated on the left. (**A**,**E**,**I**,**M**) The difference in egg volume (white dotted line). Yellow dotted line: animal pole. (**B**,**F**,**J**,**N**) The differences in yolk extension (YE) length and yolk ball’s (YB) largest diameter. Red line: wildtype YE length extrapolated to embryos with different genotypes. No differences were observed in larval development at 48 hpf and 168 hpf. The images are representative of the results observed in at least 10 embryos per experimental group. Scale bars represent 500 µm in 4 hpf, 24 hpf and 168 hpf photographs and 1000 µm in 48 hpf photographs. (**Q**) Relative egg volume at 4 hpf. The values are expressed as the percentage of the volume of wildtype eggs (*n* = 20). (**R**) YE/YB ratio at 24 hpf (*n* = 3–6). (**S**) *Cyp1b1* mRNA levels in zebrafish at 48 hpf and 168 hpf. Pools of 45 F4 zebrafish at 48 and 168 hpf were used to calculate *cyp1b1* mRNA levels by RT-qPCR. The values are expressed as relative expression levels normalized to the wild type. Three independent experiments carried out in triplicate were used for calculation of mean expression values in each sample. Asterisks indicate statistical significance compared to the wild type, *p* < 0.05 (*), *p* < 0.001 (***).

Next, we analyzed the early phenotypes of the established *cyp1b1*-KO zebrafish line, i.e., the offspring of young (<six months) inbred F3 zebrafish (F4). At 4 hpf, all the *cyp1b1*-KO embryos presented a reduced egg volume (Figure 4A,E) that was 60% of that of the wild type (Figure 4Q); at 24 hpf, they exhibited developmental delay characterized by reduced somite number (Figure S5), decreased yolk extension (YE, the posterior elongated region of the yolk cell that forms during the segmentation period) length and a similar yolk ball’s (YB) largest diameter (Figure 4B,F), resulting in a significantly lower YE/YB ratio compared to wildtype embryos (0.5 vs. 0.95, respectively; Figure 4R).

Normally, at this stage (24 hpf), the YE equals the greatest diameter of the YB (Figure 4B), and the relative length of these two parameters is useful for zebrafish staging [49]. Compared with F0 crispants, F4 *cyp1b1*-K0 embryos at 48 hpf and 168 hpf did not present significant ocular or craniofacial defects (Figure 4G,H vs. Figure 4C,D), indicating that the initial developmental delay is compensated by the end of the pharyngula period (48 h) [49]. In addition, no significant histological differences were observed in the head (Figure S6A,B) and in glaucoma-related structures, i.e., in dorsal and ventral anterior chamber angles (Figure S6B–G and Figure S6C–H, respectively), retina (Figure S6D–I) and cornea (Figure S6E–J) of these F4 larvae at 168 hpf.

Because differences in egg volume are unlikely to be affected by the embryo’s genotype, we evaluated the possible dependence of egg volume on the maternal *cyp1b1* genotype (Figure 4I–P). Heterozygous embryos obtained from young (<six months) *cyp1b1*^-/-^ female zebrafish also presented reduced egg volume (60% of that of the wild type, Figure 4I,Q), and they also presented early developmental retardation (Figure 4J) with significant reduction of the YE/YB ratio compared with the wild type (0.8 vs. 0.95, respectively, Figure 4R), although this ratio was lower than that observed in *cyp1b1*-KO embryos obtained from KO progenitors (0.8 vs. 0.6 for +/- and -/-, respectively; Figure 4R). Interestingly, the heterozygous offspring resulting from wildtype females had normal egg volume and early embryo development (Figure 4M,N) as well as an unaltered YE/YB ratio (Figure 4R). These results evidence the maternal inheritance of these defects, indicating correlation with oocyte *cyp1b1* mRNA levels. To confirm this hypothesis, embryo and larva *cyp1b1* mRNA was quantitated using RT-qPCR. We found that the offspring of *cyp1b1*-KO progenitors presented a significant reduction in mRNA at both 48 hpf and 168 hpf (27.6% and 19.1% of that of the wild type, respectively; Figure 4S). Interestingly, the heterozygous progeny of *cyp1b1*-KO males and wildtype females presented *cyp1b1* mRNA levels similar to those of wildtype embryos at 48 hpf, but they were reduced to around 35.6% at 168 hpf (Figure 4S), suggesting the presence of maternal *cyp1b1* mRNA in early stages of zebrafish development and its role in egg volume and embryo development at least up to the pharyngula stage. Consistent with these ideas, the heterozygous offspring of *cyp1b1*-KO females and wildtype males presented *cyp1b1* mRNA levels that were approximately 35% of those of wildtype embryos at both 48 hpf and 168 hpf (Figure 4S) in addition to a reduced egg volume and YE/YB ratio, as descried earlier (Figure 4I,J).

**Figure 5:**
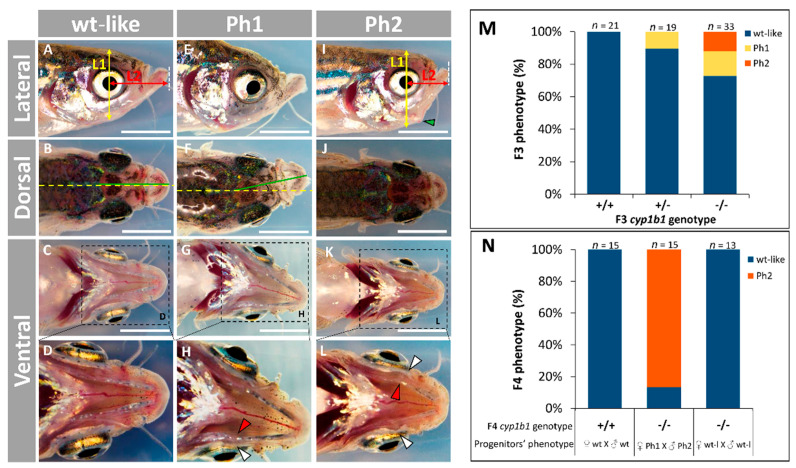
Adult (four months) craniofacial phenotypes in the established *cyp1b1*-KO zebrafish line (F3 and F4). (**A**–**L**) Phenotypes of homozygous (-/-) *cyp1b1*-KO F3 zebrafish. (**A**) Lateral craniofacial shape was assessed by the L1/L2 ratio, where L1 (yellow line) is the dorsoventral head length centered in the pupil and L2 (red line) is the anteroposterior head length from the center of the pupil to the end of the lower jaw (white dashed line). (**B**) Jaw asymmetry was evaluated measuring the dorsal angle (da) formed between the longitudinal central body axis (yellow dashed line) and an imaginary line linking the supraoccipital axis and the joining point of the two Meckel’s cartilages (green line). (**C**,**D**) Ventral morphology of wild type-like head. (**E**–**H**) Phenotype 1 (Ph1) was characterized by jaw asymmetry, i.e., da > 5°, which is clearly visible in (**F**). (**I**–**L**) Phenotype 2 (Ph2) was identified by altered craniofacial shape defined as the L1/L2 ratio > 1.87, which is 15% higher than the mean value (1.63) in wildtype zebrafish. White arrowhead: altered quadrate bone. Red arrowhead: altered palatoquadrate bone. (**M**) Percentage of F3 craniofacial phenotypes. (**N**) Percentage of F4 craniofacial phenotypes. F3 *cyp1b1*-KO siblings with mutant or wild type-like phenotypes were inbred to obtain the F4 KO progeny (-/-). Wildtype (+/+) F3 progenitors were crossed in parallel as a control. The images in (**A**–**L**) are representative of the results observed in 33 homozygous (-/-) *cyp1b1*-KO F3 zebrafish. Scale bar = 2.5 mm.

To identify possible adult phenotypes resulting from *cyp1b1* LoF, we obtained 200 F3 juvenile (one-month) zebrafish that presented genotype proportions that followed the expected Mendelian ratios. We selected 33 mutant homozygotes (-/-) that were bred and evaluated for the presence of both macroscopic and histological alterations. In addition, 19 heterozygous (+/-) and 21 wildtype siblings (+/+) were also selected and evaluated in parallel as controls. The adult (four months) *cyp1b1*-KO zebrafish were classified into two abnormal craniofacial phenotypes based on lateral cranial shape and jaw asymmetry (Figure 5A,B).

Phenotype 1 (Ph1; Figure 5E–H) was characterized by variable degrees of jaw asymmetry that was clearly observed in dorsal view (Figure 5F). Altered lateral (Figure 5I) and ventral (Figure 5K–L) craniofacial shape defined phenotype 2 (Ph2; Figure 5I–L). Detailed ventral observation of this phenotype revealed that both quadrate and palatoquadrate cartilages were curved outward (Figure 5K,L, white and red arrowheads, respectively) compared wildtype zebrafish (Figure 5C,D). Dorsal examination of Ph2 did not show significant alterations (Figure 5J). Unexpectedly, Ph1 was observed in approximately 10% of the heterozygous (+/-) zebrafish and this phenotype increased to 15.1% in their mutant homozygous (-/-) siblings (Figure 5M). In addition, around 16.6% of *cyp1b1*-KO zebrafish presented Ph2 (Figure 5M), summing a total of approximately 32% mutant phenotypes in the F3 generation. To further assess the inheritance of the observed phenotypes, F3 *cyp1b1*-KO Ph1 females were inbred with F3 *cyp1b1*-KO Ph2 males. Fifteen KO F4 zebrafish from two independent crosses were randomly selected to evaluate their adult (12 months) phenotypes. We observed that 86.6% of the offspring showed the parental Ph2 (with 13.4% wild type-like), but none of the zebrafish presented Ph1 (Figure 5N), which represents a three-fold increase of craniofacial phenotypes in this generation. In addition, F3 *cyp1b1*-KO siblings with wild type-like phenotypes were also mated in parallel and no abnormal phenotypes were observed in their F4 KO progeny (Figure 5N). As expected, the offspring of wildtype (+/+) F3 progenitors presented normal phenotypes (control). These results further support the existence of incomplete penetrance and variable expressivity in the craniofacial alterations associated with *cyp1b1* LoF as well as a role for the genetic background in these phenomena.

**Figure 6:**
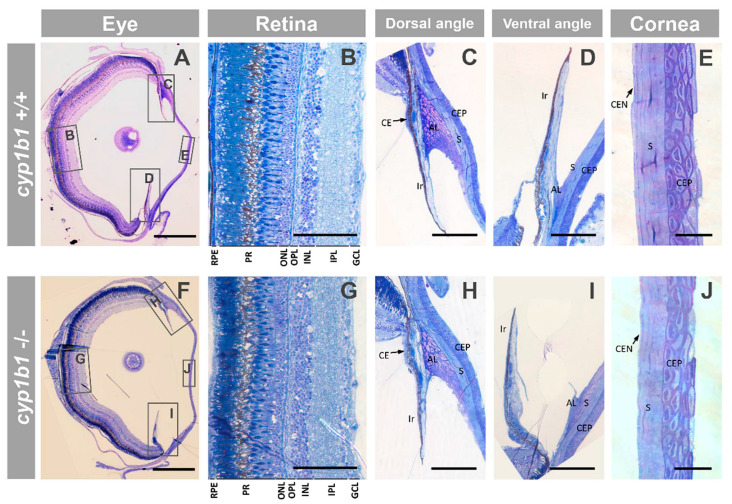
Ocular histology of adult (seven months) *cyp1b1*-KO zebrafish with Ph2 craniofacial alterations (F3). (**A**–**J**) Semithin (500 nm) tissue sections were stained with toluidine blue. The squares and rectangles indicate the areas of the images magnified in the indicated panels. No significant differences were observed between the eyes of wildtype and *cyp1b1*-KO zebrafish siblings. Scale bar in (**A**,**F**): 500 μm. Scale bar in (**B**–**D**,**G**–**I**): 100 μm. Scale bar in (**E**,**J**): 25 μm. RPE: retinal pigment epithelium; PR: photoreceptors; ONL: outer nuclear layer; OPL: outer plexiform layer; INL: inner nuclear layer; IPL: inner plexiform layer; GCL: ganglion cell layer; CE: ciliary epithelium; AL: annular ligament; CEP: corneal epithelium; CEN: corneal endothelium; S: stroma. The images are representative of the results observed in three fishes of each genotype. Three tissue sections per eye were analyzed.

Histological analysis of semithin ocular sections obtained from adult (seven months) F3 *cyp1b1*-KO zebrafish with craniofacial Ph2 alterations did not reveal significant global alterations (Figure 6A,F). In addition, the retina- (Figure 6B,G) and glaucoma-related ocular tissues, i.e., anterior chamber angles (Figure 6C,D,H,I) and cornea (Figure 6E,J), were similar in *cyp1b1*-KO and wildtype siblings.

### 2.3. Comparison of Gene Expression Profiles of Cyp1b-KO and Wildtype Zebrafish

To investigate gene expression changes associated with *cyp1b1* LoF, we performed comparative whole-transcriptome sequencing of 168 hpf *cyp1b1*-KO and wildtype zebrafish larvae of the same age. The mutant larvae were obtained by inbreeding F3 *cyp1b1*-KO progenitors with the most penetrant phenotype (Ph2). To reduce individual variability, we pooled 45 larvae in each sample. Two independent biological replicas of each experimental group (*cyp1b1*-KO and wild type) were analyzed. The edgeR package implemented in Rstudio was used to detect differentially expressed genes (DEGs) in the *cyp1b1*-KO larvae compared with wildtype larvae. From the total of 33,537 analyzed genes, 4947 unmapped or low-expressed genes with zero read counts in all the samples were excluded from the analysis, leaving 28,590 genes for statistical analyses. The correlation matrix of all the samples using Pearson’s coefficient supported the similarity between replicas (Figure S7A). Log fold change (FC) and average log counts per million (CPM) were plotted (MA plot) to assess transcriptional bias between *cyp1b1*-KO and wildtype transcriptomes. Most of the points on the Y-axis were located at 0 (Figure S7B), indicating that the parameters used to identify differentially expressed genes in the dataset were appropriate. Consistency of differentially expressed gene patterns in the different experimental replicas was also evaluated by a heatmap of hierarchical clustering. The results showed similar DEG clusters between replicas of the same experimental group, indicating that most of the identified gene expression patterns are reproducible and consistent (Figure S7C).

**Figure 7:**
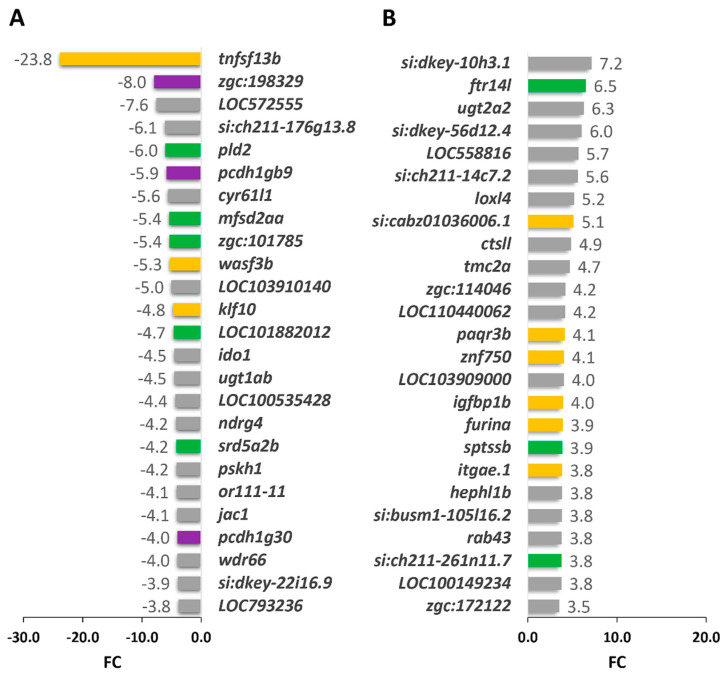
Top 25 DEGS in *cyp1b1*-KO vs. wildtype seven-dpf zebrafish larvae. (**A**) Down- and (**B**) upregulated genes identified by high-throughput RNA sequencing with significant differences in the comparison. Yellow bars: development signaling genes. Green bars: lipid metabolism genes. Purple bars: extracellular matrix and cell adhesion genes. Grey: down- and upregulated genes from other functional pathways.

The filtering of DEGs with an absolute log_2_ fold change of at least 1 and a *p*-value of <0.05 identified 451 genes (185 up- and 266 downregulated; Table S1). These genes were included in the functional pathway analysis. The top 25 up- and downregulated genes are listed in Figure 7. Interestingly, nine of these highly altered genes were found to be involved in development signaling, seven genes participated in lipid metabolism, and three genes played a role in the ECM and cell adhesion.

#### 2.3.1. Functional Enrichment Analysis of DEGs

Next, the whole group of 451 DEGs with fold change enrichment of >±2 was subjected to functional enrichment analysis using the David bioinformatic webtool (https://david.ncifcrf.gov/) (accessed on 1 December 2020) to identify genes overrepresented in different pathways, biological processes and molecular functions. Seven statistically significant (*p*-value < 0.05) Kyoto Encyclopedia of Genes and Genomes (KEGG) metabolic-related pathways were observed to be affected by *cyp1b1* LoF (Table 1): steroid hormone biosynthesis, PPAR signaling pathway, retinol metabolism, drug xenobiotic metabolism and cytochrome P450 xenobiotic metabolism and primary bile acid biosynthesis and steroid biosynthesis.

Biological process analysis (Table S2) showed 17 significantly enriched processes that can be classified into four broad functionally related groups: (i) ECM and cell adhesion (proteolysis, which included several ECM metalloproteases, cell adhesion and homophilic cell adhesion via plasma membrane adhesion molecules, with 39 genes), (ii) cell growth and proliferation (regulation of cell proliferation and regulation of transcription from RNA polymerase II promoter (18 genes)), (iii) lipid metabolism and metabolic processes (lipid metabolic process and lipid transport, oxidation–reduction processes, intracellular sequestering of iron ions and iron ion transport, which included several cytochrome P450 genes (38 genes)) and (iv) inflammation and immunity (response to lipopolysaccharides, inflammatory response, neutrophil chemotaxis and activation, response to cytokines, response to bacteria and immune response, encompassing 22 genes).

The molecular function analysis of DEGs (Table S3) corroborated the results described and identified terms mainly related to two biological processes determined in the previous analysis: (i) peptidase and hydrolase activities associated with ECM metalloproteinases and (ii) metabolic-related monooxygenase, oxidoreductase and heme-binding activities related with cytochrome P450 genes and lipid-binding functions. Interestingly, five of the differentially expressed cytochrome genes were downregulated, and only one of them, *cyp24a1*, was upregulated, suggesting that it could participate in the genetic compensation of *cyp1b1* LoF. In summary, functional enrichment analysis identified significant DEGs involved in extracellular matrix and cell adhesion, lipid metabolism (retinol, steroids and fatty acids), cell growth and proliferation and inflammation pathways.

#### 2.3.2. Validation of RNA-Seq Results

**Figure 8:**
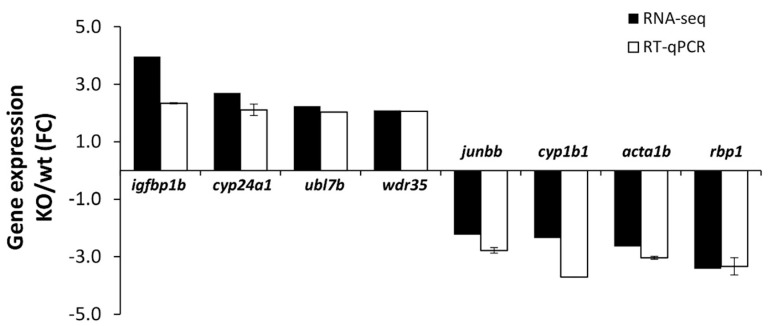
Confirmation by RT-qPCR of the differential expression of the selected genes identified in the RNA-seq analysis of *cyp1b1*-KO zebrafish. RT-qPCR was carried out in triplicate.

To confirm differential gene expression by RT-qPCR, we selected *cyp1b1* and seven representative genes of the main functional groups identified (Table 1, Tables S2 and S3), which could potentially contribute to the craniofacial phenotypes because they are or may be involved in the metabolism or transport of morphogens (*ubl7b*, *cyp24a1* and *rbp1*) or may play a role in processes such as cellular growth, migration and differentiation (*igfbp1b*, *acta1b*), signal transduction or regulation of genes involved in embryo development (*wdr35*, *junbb*) (Figure 8). Quantitative PCR confirmed an approximately three-fold *cyp1b1* downregulation (Figure 8), as previously observed (Figure 2D). The rest of the genes also presented a good correlation with the transcriptome data (Figure 8).

## 3. Discussion

*CYP1B1* LoF mutations are the main identified genetic cause of CG; however, the pathogenic mechanisms are not clear. To the best of our knowledge, this is the first *cyp1b1*-KO zebrafish model generated to analyze the mechanisms underlying *cyp1b1* LoF. The CRISPR/Cas9 *cyp1b1*-KO zebrafish line carried the c.535_667del133 deletion. RT-qPCR demonstrated a remarkable reduction in *cyp1b1* mRNA. In addition, this mutation was predicted to lead to a frameshift (p.(His179Glyfs*6)) and to a truncated cyp1b1 enzyme translated from residual mutant mRNA. The truncated protein lacks important functional domains, including the enzyme active center, which is located downstream of the premature termination codon. Altogether, these data support that the obtained mutation results in a complete *cyp1b1* LoF.

Approximately 25% of F0 *cyp1b1* crispant larvae presented variable microphthalmia and lower jaw underdevelopment at 144 hpf. These early defects might have been due to disrupted migration of neural crest-derived cells, which are involved in cranial and jaw morphogenesis [50]. Consistent with this idea and with our results, cyp1b1 has been described to be expressed in the developing eye and pharyngeal arches both in zebrafish [45] and in chicken [51] embryos, and zebrafish *cyp1b1* knockdown affects the development of neural crest cell-derived tissues in zebrafish, resulting in early mild ocular defects [47]. In contrast, the established *cyp1b1*-KO zebrafish line did not manifest these early phenotypes, although at 24 hpf, all the embryos presented two new features: egg volume reduction and transitory developmental delay that completely recovered at 48 hpf. Accordingly, craniofacial and ocular developmental delay observed in zebrafish *cyp1b1*-knockdown in the first 48 hpf also recovers by 96 hpf [45]. Interestingly, the egg and growth abnormalities in the *cyp1b1*-KO zebrafish line were exclusively observed in the offspring of *cyp1b1*-KO females and correlated with *cyp1b1* mRNA levels during early embryonic development, demonstrating their maternal inheritance and suggesting the participation of maternal *cyp1b1* mRNA in early embryo development. Remarkably, the early morphological phenotypes were absent in the established *cyp1b1*-KO zebrafish line, which might be explained by lethality and/or compensating mechanisms. *Cyp1b1* LoF may be lethal in F0 zebrafish with susceptible genetic backgrounds, leading to selection of animals with compensating genetic backgrounds. Consistent with this hypothesis, we did not observe morphological defects among adult F0 crispants (>one year), suggesting that phenotypically affected larvae probably died due to feeding limitations associated with craniofacial defects. In addition, phenotypic differences between F0 crispants and established KO zebrafish lines are not uncommon [52,53,54] and may result from functional replacement of the deactivated gene by functionally related paralog or non-paralog compensatory genes [53]. These compensatory genes may be more easily upregulated in stable genetically engineered KOs than in microinjected F0 mosaic KOs [53]. Moreover, mutations that activate NMD mechanisms, such as those present in our *cyp1b1*-KO zebrafish line, are more prone to triggering compensatory mechanisms [54,55] than posttranscriptional interferences, such as those produced by MO knockdown.

The main phenotype detected in the *cyp1b1*-KO zebrafish line comprised variable adult-onset jaw and craniofacial alterations (increased head height and reduced jaw length), suggesting that disrupted ECM alterations may underlie these defects. Consistent with this hypothesis, defects in ECM remodeling, more than deposition failures, have been proposed to cause progressive TM atrophy associated with fragmentation and irregular distribution of collagen fibers present in aging *Cyp1b1*-KO mice and absent in young animals (< two weeks old) [34]. We were not able to determine the exact age onset of the craniofacial phenotype. Further work is required to determine when these defects start to manifest. The adult craniofacial alterations observed in our *cyp1b1*-KO zebrafish line also presented incomplete penetrance and variable expressivity characterized by uni- (Ph1) or bilateral (Ph2) jaw shortening. Inbreeding increased the penetrance from 26.6% to 86.6%, indicating that the phenotype is strongly influenced by the genetic background. The typical human phenotype associated with *CYP1B1* LoF, i.e., PCG, also presents phenotypic variability [56] and incomplete penetrance [57], illustrating that although the phenotypes are different in these two species, they are also highly influenced by the genetic background. Another interesting parallelism between this *cyp1b1* LoF zebrafish model and human CG [12] is the unexpected presence of abnormal phenotypes in some heterozygotes, which again indicate the role of modifiers in these phenotypes. In contrast to humans, we did not observe ocular glaucoma-related histological defects associated with complete *cyp1b1* LoF in zebrafish, which might be due to developmental species differences and shows that zebrafish are not adequate to model *cyp1b1*-associated glaucoma. In accordance with our results, 48-hpf zebrafish embryos with MO *cyp1b1* knockdown did not present glaucoma; they only manifested mild ocular phenotypes that recovered by the larval stage [47] and presented minimal effects on zebrafish craniofacial development at 96 hpf [45]. Nevertheless, microinjection of human wildtype *CYP1B1* mRNA but not of LoF mutant versions reproduces phenotypes resulting from *cyp1b1* overexpression in zebrafish larvae [45], showing the functional equivalence between the human and zebrafish ortholog proteins. Mammalian species such as mice or even other species with ocular developmental pathways phylogenetically closer to those of humans may be needed to develop appropriate CG models. In this regard, *Cyp1b1*-KO mouse models show subtle iridocorneal angle abnormalities also dependent on modifier factors such as *Tyr* deficiency, but these defects result in undetectable [32] or modest intraocular pressure elevation [33]. Interestingly, *Tyr* is not a modifier of the PCG phenotype in humans [58], supporting that *CYP1B1*-associated phenotypes are species-specific. Keeping in mind these limitations, the zebrafish may provide valuable information to determine the precise biological functions of *cyp1b1* as well as to understand the general pathogenic processes underlying *cyp1b1* LoF.

To characterize the molecular basis of the phenotypes associated with *cyp1b1* LoF, we performed a transcriptomic analysis in the offspring (seven dpf) of *cyp1b1*-KO zebrafish with craniofacial defects. The functional enrichment analysis of DEGs identified a consistent alteration of genes involved in three biological processes that could be directly related to the observed phenotypes: (i) the ECM and cell adhesion, (ii) the regulation of cell proliferation and (iii) lipid metabolism (retinol, steroids and fatty acids). In addition, metabolic-related oxidation–reduction processes, which included many cytochrome P450 genes, and immune response and inflammation were also significantly enriched in our analysis.

In the first group, we found altered expression of a repertoire of matrix metalloproteinase (MMP)-encoding genes that may disrupt ECM assembly and remodeling, playing a direct role in adult and early craniofacial phenotypes observed in *cyp1b1*-KO zebrafish. Some of these MMPs participate in neural crest-derived cell migration (*ADAMTS20A* or *LOC101886654*) [59], regulate fibronectin levels in zebrafish (*mmp11b*) [60] or break down elastin and other proteins (*cela1.3*, a serine-type endopeptidase orthologous to the human chymotrypsin-like elastase 1 or *CELA1*) [61]. Similarly, the identification of cell adhesion DEGs, such as those encoding protocadherins (*Pcdh1g30*, *Pcdh1g3*, *Pcdh1gb9*, *Pcdh1g2* and *Pcdh1g26*), desmosomal proteins (desmoglein (*Dsg2.1*) and desmocollin (*Dsc2l*)) and periostin (*Postna*) indicate possible dysregulation of developmental signaling and developmental processes, including morphogenesis [62,63]. In fact, *Postna* modulates ECM organization [64] and is involved in ocular developmental defects observed in the *Cyp1b1*-KO mice [33], and MO-mediated *dsg2.1* knockdown is associated with head development disruption [65].

Functionally enriched DEGs playing a role in cell proliferation pathways and craniofacial morphogenesis suggested an alteration in development signaling in the *cyp1b1*-KO zebrafish that might also contribute to the craniofacial phenotypes observed in adult mutant zebrafish and maybe in F0 crispant larvae. Among these genes, we found members of the c-Jun/AP-1 (*junba* and *junbb*) canonical Wnt (*wnt9b*) signaling pathways, indicating that those members were altered. Interestingly, *wnt9b* knockdown produces jaw and craniofacial defects in zebrafish larvae [66]. On the other hand, downregulation of some genes of this group (*grhl3*, *furina*, *ahrra* and *cdk6)* leads to craniofacial maldevelopment in different animal models [67,68,69]. Three of these genes (*grhl3*, *furina* and *ahrra*) were upregulated in our animal model, suggesting they might participate in possible genetic compensation of *cyp1b1* LoF. Additional downregulated genes such as *fosl1a* and *relb* participate in bone matrix remodeling [70] and osteoclast differentiation [71], respectively.

Regarding lipid metabolism, we identified four DEGs (*rbp1*, *rbp2b*, *ugt2a2* and *ugt1ab*) involved in retinol transport and metabolism [72], suggesting that retinol metabolism alteration might be an additional mechanism contributing to the observed phenotypes. Retinoid signaling plays a key role in embryonic development of different organs, including the eye [73], and alteration of this pathway may disrupt migration of cranial neural crest cells, leading to ocular and craniofacial defects [74,75,76,77], similar to those observed in our *cyp1b1*-KO zebrafish line. In addition, and consistently with this idea, cyp1b1 has been described to metabolize retinol to retinaldehyde and then to retinoic acid (RA) in vitro [3,51], and treatment of zebrafish with exogenous RA results in prognathic jaw development, while inhibition of endogenous RA decreases head height [78], resembling the phenotypes observed in the *cyp1b1*-KO zebrafish. Further investigations are necessary to elucidate the involvement of retinoids in our *cyp1b1*-KO zebrafish model. Genes involved in steroid hormone biosynthesis and functionally related with *cyp1b1* were also differentially expressed in the *cyp1b1*-KO zebrafish, although only three of them (i.e., *cyp24a1*, *ugt2a2* and *hsd11b2*) were upregulated, indicating their possible participation in *cyp1b1* LoF compensation. *Cyp24a1* participates in vitamin D hydroxylation and fatty acid omega oxidation and it is associated with hyperlipidemia in rats [79]. Alteration in lipid metabolism is further supported by the identification of several DEGs of the lipid metabolism-modulating PPAR signaling pathway [80], including, for instance, *cyp7a1* and *cyp8b1*, which are involved in bile acid biosynthesis [81]. In line with our findings, *Cyp1b1*-KO mice present PPAR pathway dysregulation [41], although some key genes followed different trends in our study. For instance, *igfbp1*, a regulator of liver fatty acid homeostasis, was overexpressed in our study and downregulated in KO mice. *Igfbp1* expression is affected by diet and sex [41,43], therefore, differences in these variables may explain the discrepancy. The finding of altered expression of lipid metabolism genes and lipid composition in *Cyp1b1*-KO mice is also consistent with our results [41,43,82]. Similarly interesting is the identification of differentially expressed redox genes, including several upregulated cytochrome P450 family members (e.g., *cyp24a1*), suggesting that they may compensate, at least partially, *cyp1b1* LoF. Finally, inflammation pathways were also affected in *cyp1b1*-KO zebrafish, which is in line with the inflammatory response inhibition reported in *Cyp1b1*-KO mice [39]. Alteration in inflammatory pathways in the *cyp1b1*-KO zebrafish is supported by the reported roles of this cytochrome in inflammation. In fact, *cyp1b1* is induced in response to inflammation [83] and, along with *Cyp1a1* and *Cyp1a2*, it participates in lipid mediator pathways that regulate neutrophilic inflammation in mice [42]. Further work is required to confirm the status of inflammatory pathways in the zebrafish *cyp1b1* mutant.

One limitation of the transcriptomic analysis presented herein is that it was performed using whole larvae, but the main phenotypes were limited to adult craniofacial structures. Therefore, RNA-seq of isolated adult craniofacial tissues is required to further characterize and refine DEGs involved in these phenotypes. Moreover, ultrastructural and lipidomic analyses are needed, respectively, to confirm the presence of ECM and lipid alterations in this *cyp1b1*-KO zebrafish line.

## 4. Materials and Methods

### 4.1. Zebrafish Embryo Management 

Zebrafish embryos were maintained at 28 °C in a fish water medium (5 mM NaCl, 0.17 mM KCl, 0.33 mM CaCl_2_, 0.33 mM MgSO_4_ and 0.0001% methylene blue, pH 7.2). For imaging, adult species and larvae were anaesthetized with 0.02% tricaine methanesulfonate (MS 222, SigmaAldrich, St. Louis, MO, USA) and immobilized in Petri dishes with a 3% agarose mold or in a 2% methylcellulose solution, respectively. The Animal Research Committee of the University of Castilla–La Mancha approved zebrafish husbandry and experiments (approval number PR-2017-01-19).

### 4.2. Cas9 Gene Editing 

Alt-R CRISPR-Cas9 guide RNA (https://eu.idtdna.com/site/order/designtool/index/CRISPR_CUSTOM, Integrated DNA Technologies) (accessed on 5 June 2019) and CHOPCHOP V.3 programs (http://chopchop.cbu.uib.no, access on 5 June 2019) were employed to select zebrafish *cyp1b1* targets and to design crRNA. CRISPR RNA putative off-target sequences and maximum on-target efficiency were evaluated with the CRISPR-Cas9 guide RNA design checker (https://eu.idtdna.com/site/order/designtool/index/CRISPR_SEQUENCE, Integrated DNA Technologies) (accessed on 5 June 2019). Trans-activating CRISPR RNA (tracrRNA) and crRNAs were purchased from Integrated DNA Technologies. Deletions of approximately 100 bp were generated in exon 1 using crRNA 1.1 (GACACCACTAAATACCGGTG-CGG) and crRNA 1.2 (TTCACCAAAACAGTCGGAGC-CGG). For Cas9/crRNA/tracrRNA microinjections, crRNA pairs (36 ng/μL final concentration of crRNA 1.1 and crRNA 1.2) and tracrRNA (67 ng/μL final concentration) were mixed, incubated for 5 min at 95 °C and cooled at room temperature. The Cas9 protein (Alt-R^®^ CRISPR-Cas9 at 250 ng/μL, IDT) and the tracrRNA/crRNAs complex were mixed and incubated for 10 min at 37 °C. A Femtojet 5247 microinjector (Eppendorf) and a Nikon DS-Ri2 stereomicroscope were employed to inject Cas9/tracrRNA/crRNAs complexes (3 nL) into the animal pole of zebrafish embryos at the one-cell developmental stage (50–250 embryos/experiment). The negative control consisted of embryos injected only with Cas9/tracrRNA. The experiments were repeated independently at least three times and different progenitors were selected for each experiment.

### 4.3. Genotyping

Genomic DNA of zebrafish larvae or adult tail biopsies was extracted by alkaline lysis (a method for high-throughput PCR-based genotyping of larval zebrafish tail biopsies, Robert N. Wilkinson, Biotechniques, 2018). The samples were incubated for 30 min at 95 °C in 20 μL lysis buffer (KOH 1.5 M and EDTA 10 mM) and neutralized with 20 μL neutralization buffer (TRIS HCl 2M). The presence of deletions in exon 1 was analyzed by PCR using the following primer pairs: CRISPR CYP1B1 M2 F1 SEQ, 5’-GCAGAGCACCGTCAGAAATT-3′/ CRISPR CYP1B1 M2 R1 SEQ, 5’-ATGAACGCGCAAAACTCCTT-3’. Thermocycling for both amplicons consisted of 95 °C for 10 min followed by 30 cycles of 95 °C for 30 s, 61.1 °C for 30 s and 72 °C for 30 s. Then, the samples were analyzed using 1% agarose gel electrophoresis.

### 4.4. RT-qPCR

Quantitation of *cyp1b1* mRNA or of selected DEGs relative to *ef1a* mRNA was determined using the 2^-ΔΔCt^ method [84] using the primer pairs indicated in Table S4. Pools of 50 zebrafish larvae (48 hpf and 168 hpf, approximately 30 mg/pool) were used to extract total RNA using the TRI reagent (SIGMA) following the manufacturer’s instructions. The first strand of cDNA was synthesized from purified total RNA (approximately 1.5 μg in 20 μL) using a RevertAid First Strand cDNA Synthesis Kit (#K1622, Thermo Fisher Scientific, Waltham, MA, USA). PCR analysis was carried out in total reaction volumes of 10 μL containing 2 μL template cDNA, 5 μL Power SYBR Green PCR Master Mix (Thermo Fisher Scientific) and 200 nM of each primer. Thermocycling was carried out in an ABI PRISM 7500 Fast real-time PCR system (Life Technologies, Foster City, CA, USA) and consisted of 95 °C for 10 min followed by 40 cycles of 15 s at 95 °C for 60 s and 60 °C for 40 s (combined annealing and extension). The negative controls consisted of all component reactions without template cDNA. PCR reactions produced single bands in agarose electrophoresis.

### 4.5. Fluorescent In Situ Hybridization

The templates for *cyp1b1* riboprobes were amplified from a commercial *cyp1b1* cDNA clone (#8146986, Source Bioscience, Nottingham, UK) and cloned into the pCRII-TOPO plasmid (ZFcyp1b1UPNotI: 5′-GTATCCAGAAATCCAGAAGCGTCTCC-3 and ZFcyp1b1DWSacI: 5′-CTTGGAGTCTGAGATGTTCCTACCAA-3′). Sense and antisense riboprobes were transcribed from the linearized plasmid using either T7 or Sp6 RNA polymerase and were fluorescently labeled using a FISH Tag RNA Green Kit (Life Technologies). Dechorionated embryos were fixed overnight at 4 °C in 2% sweet paraformaldehyde (2% PFA, 4% sucrose, 10 mM phosphate-buffered saline (PBS), pH 7.3). Then, they were dehydrated and stored in 100% methanol. A hybridization mix (50% (*v*/*v*) deionized formamide, 5× saline sodium citrate buffer (750 mM NaCl, 75 mM Na_3_ citrate (SSC), 5 mg/mL tRNA, 50 μg/mL heparin and 0.1% Tween-20, pH 6.0)) was used to prehybridize proteinase K-permeabilized embryos at 55 °C for 4 h. Hybridization was carried out with a fluorescent riboprobe (100 ng) overnight. SSC-washed embryos were oriented and mounted in the Fluorescent Mounting Medium and visualized using an LSM800 Zeiss confocal microscope. The fluorescence emitted by Alexa 488-conjugated riboprobes (495–529 nm) and embryo autofluorescence (553–677 nm) was registered. The ZEN software (Carl Zeiss, Jena, Germany) was employed to obtain maximum intensity projections of Z-Stack.

### 4.6. Examination of Mutant Phenotypes by Light Microscopy

Egg volume was assessed at 4 hpf in a Petri dish with an E3 medium. Embryo and larval phenotypes were evaluated using dechorionated zebrafish larvae at 24 hpf, 48 hpf, 144 hpf and 168 hpf, handled in methylcellulose. The specimens were observed using a Nikon DS-Ri2 microscope and the egg radius and embryo length were measured with the NIS-Elements BR 4.50.00 software (Nikon, Tokyo, Japan). The egg volume was calculated using the sphere volume formula (4/3 πr^3^, where r is the egg radius). The eyes of seven-month-old adult fish and larvae at seven dpf were fixed in 2.5% glutaraldehyde/4% paraformaldehyde in 0.1 M Millonig’s phosphate buffer (PBM, pH 7.4) overnight at 4 °C. Then, the samples were washed in PBM and post-fixed in 1% osmium tetroxide for 1 h at room temperature. After further PBM washing steps, ascending grades of acetone (30–100%) were used for tissue dehydration. Finally, the samples were embedded in araldite. Toluidine blue (1% in 1% sodium tetraborate) was used to stain semithin (0.5 μm) tissue sections. Optical microscopy was carried out with a Nikon Eclipse-Ti microscope.

### 4.7. RNA Preparation and Transcriptome Analysis

Pools of 45 zebrafish larvae (seven dpf, approximately 30 mg) were homogenized using the TRI reagent (SIGMA) and following the manufacturer’s instructions. After Trizol extraction, total RNA (14.5–18.9 μg) was further purified using RNAeasy columns (Qiagen, Germantown, MD, USA) and treated with DNAse to remove contaminating DNA. The quality of RNA samples was assessed both by spectrophotometry (NanoDrop 2000, Thermo Fisher Scientific) and by agarose gel electrophoresis. RNAseq was carried out using MacroGen Next Generation Sequencing Division (Macrogen, Korea) using the Illumina HiSequation 2000 platform. Complementary DNA libraries were constructed using an Illumina TruSeq RNA library preparation kit (Illumina). The resulting libraries were sequenced with NovaSeq6000 Sequencing System (Illumina) (2X150) 50M reads. The quality control of the sequenced raw reads was determined using the Phred score. To reduce biases in analysis, sequences with low-quality reads, adaptor sequences, contaminant DNA sequences or PCR duplicates were removed. Trimmed reads were mapped to the reference genome with HISAT2, a splice-aware aligner. GCF_000002035.6 was used as the reference genome to map sequences. Known genes and transcripts were assembled using StringTie with aligned reads. Expression profiles were represented as read counts and normalized values on the basis of transcript length and depth of coverage. Differentially expressed gene (DEG) analysis based on read count values was performed using the edgeR software package (version 3.32.1) [85]. A gene was considered downregulated if the fold change (FC) value was <−2 and upregulated if the FC value was >2. Functional annotation and gene-set enrichment analysis of DEGs were performed using a webtool for annotation, visualization and integrated discovery (DAVID, http://david.abcc.ncifcrf.gov/) (accessed on 1 December 2020) and the GO and KEGG databases.

### 4.8. In Silico Analysis 

Human and zebrafish gene comparison was performed with information from the Ensembl database (https://ensembl.org) (accessed on 12 June 2019). Protein sequence alignments were carried out with ClustalW. The variants were named using directions from Mutalyzer (https://mutalyzer.nl/) (accessed on 20 December 2019).

### 4.9. Statistics 

Either the *t*-test or the one-way analysis of variance (ANOVA) were used to perform statistical comparisons between groups. Multiple comparisons were adjusted with Bonferroni correction. The SigmaStat 2.0 software (SPSS Science Inc., Inc., Chicago, IL, USA) was employed to carry out the statistical analyses.

## 5. Conclusions

To the best of our knowledge, this is the first report of the generation and characterization of a *cyp1b1*-KO line in zebrafish. Although these mutant animals did not show glaucoma-related phenotypes, they developed adult-onset craniofacial alterations with incomplete penetrance and variable expressivity, evidencing the existence of compensatory genes and modifier factors. Identification of DEGs involved in ECM and cell adhesion and developmental signaling pathways indicates that alterations in these biological processes may underlie the observed phenotypes. The established *cyp1b1*-KO zebrafish line provides a new model with which to investigate the biological function of this gene and opens new avenues for studying the molecular mechanisms associated with CG pathogenesis.

## Figures and Tables

**Table 1:** KEGG analysis of DEGs in the *cyp1b1*-KO zebrafish line using the DAVID bioinformatic tool. The count indicates the number of genes included in each pathway.

KEGG Term	Count	*p*	Genes
Steroid hormone biosynthesis	7	<0.0001	*hsd11b2*, *cyp11c1*, *srd5a2b*, *cyp1b1*, *ugt2a2*, *ugt1ab*, *cyp7a1*
PPAR signaling pathway	6	0.006	*cpt2*, *ubb*, *aqp7*, *cyp7a1*, *pltp*, *cyp8b1*
Drug metabolism—other enzymes	4	0.023	*zgc:103601*, *ugt2a2*, *tk1*, *ugt1ab*
Retinol metabolism	4	0.028	*ugt2a2*, *si:ch1073-13h15.3*, *ugt1ab*, *zgc:109982*
Metabolism of xenobiotics by cytochrome P450	4	0.028	*gstt2*, *cyp1b1*, *ugt2a2*, *ugt1ab*
Primary bile acid biosynthesis	3	0.049	*ch25h*, *cyp7a1*, *cyp8b1*
Steroid biosynthesis	3	0.049	*cyp24a1*, *sc5d*, *cel.2*

## Data Availability

The data presented in this study are available in the article and supplementary materials.

## Supplementary Materials

Supplementary Materials can be found at https://www.mdpi.com/article/10.3390/ijms22126430/s1.
