# Supplementary material for: Null cyp1b1 Activity in Zebrafish Leads to Variable Craniofacial Defects Associated with Altered Expression of Extracellular Matrix and Lipid Metabolism Genes
Source: Int J Mol Sci. 2021 Jun 16;22(12):6430. doi: 10.3390/ijms22126430 (PMC8234340; doi:10.3390/ijms22126430)
Supplement: Supplementary file 1 [file ijms-22-06430-s001.zip › ijms-1247243-revised-suppl..pdf]

Supplemental Materials

Null *cyp1b1* activity in zebrafish leads to variable craniofacial defects associated with altered expression of extracellular matrix and lipid metabolism genes

Susana Alexandre-Moreno [1,2], Juan-Manuel Bonet-Fernández [1,2], Raquel Atienzar-Aroca [1,2], José-Daniel Aroca-Aguilar [1,2*] and Julio Escribano [1,2*]

[1] Área de Genética, Facultad de Medicina de Albacete/Instituto de Investigación en Discapacidades Neurológicas (IDINE), Universidad de Castilla-La Mancha, 02006 Albacete, Spain; Susana.Alexandre@uclm.es (S.A.-M.); JuanM.Bonet@uclm.es (J.M.B.-F.); Raquel.Atienzar@uclm.es (R.A.-A.); JoseDaniel.Aroca@uclm.es (J.-D.A.-A.); julio.escribano@uclm.es (J.E.)

[2] Cooperative Research Network on Age-Related Ocular Pathology, Visual and Life Quality (OFTARED), Instituto de Salud Carlos III, 28029 Madrid, Spain; Susana.Alexandre@uclm.es (S.A.-M.); JuanM.Bonet@uclm.es (J.M.B.-F.); Raquel.Atienzar@uclm.es (R.A.-A.); JoseDaniel.Aroca@uclm.es (J.-D.A.-A.); julio.escribano@uclm.es (J.E.)

* Correspondence: JoseDaniel.Aroca@uclm.es and julio.escribano@uclm.es

Supplemental Figures

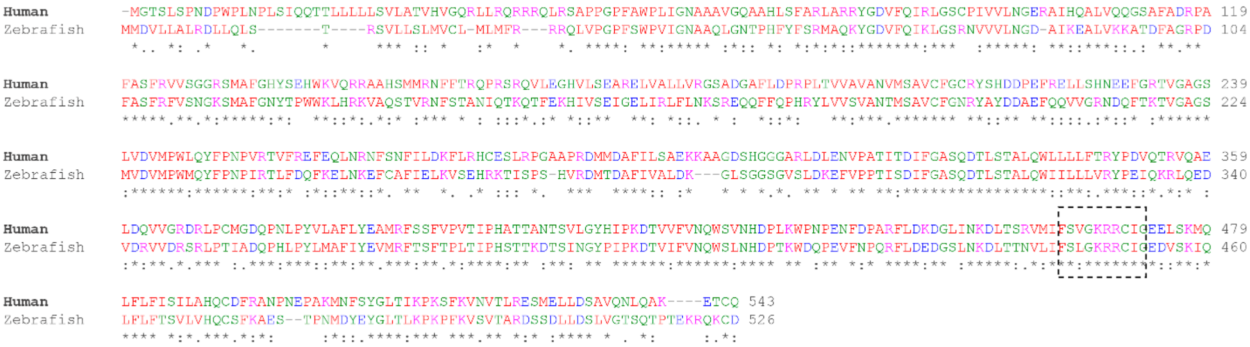

Figure S1. Amino acid sequence comparison of Human and Zebrafish cyp1b1. The alignment the human (ENSP00000478561.1 and) and zebrafish (ENSDARP00000107132.2) proteins was carried out with ClustalW (https://embnet.vital-it.ch/software/ClustalW.html). The asterisks indicate the positions where all the amino acids are identical, two vertical dots show amino acids with similar chemical properties and one dot denotes amino acid positions with weak chemical similarity. Dotted black square: Cytochrome P450 cysteine heme-iron ligand which is indicated according to the Prosite database (https://prosite.expasy.org/). The numbers correspond to amino acid positions.

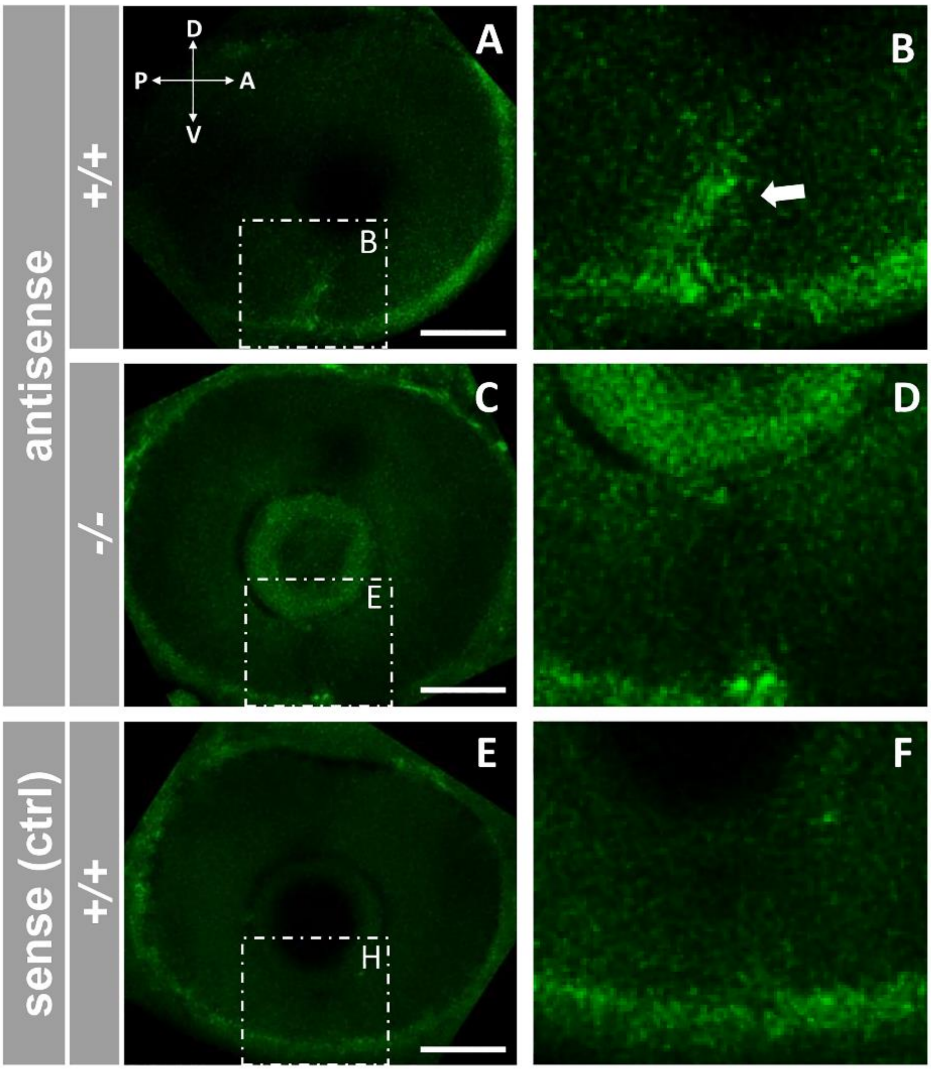

Figure S2. ***Cyp1b1*** **expression analysis by fluorescent-whole mount** ***in situ*** **hybridization in zebrafish embryo eyes at 48 hpf.** (A-D) *Cyp1b1* antisense or (E and F) sense (control) RNA probes were labelled with Alexa Fluor-488 (green). The arrow indicates specific *cyp1b1* expression in the optic fissure. The inset in (A) indicates the position of embryonic axes (D: dorsal; P: posterior; V: ventral; A: anterior). The image is representative of the result observed in 3 embryos of each genotype. Scale bar 50µm.

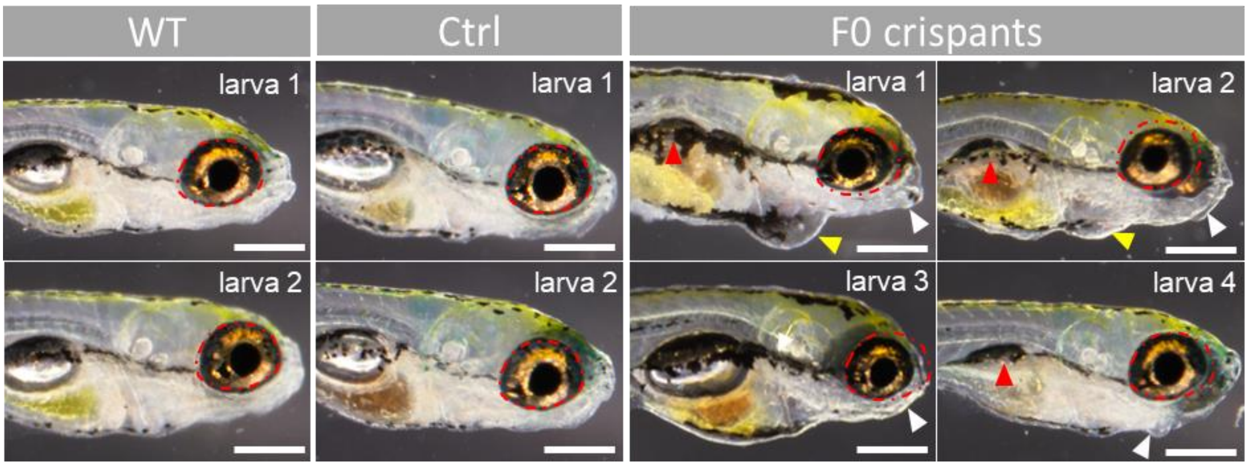

Figure S3. Phenotypic variability of c*yp1b1* F0 crispants (144 hpf) shown in Fig 3. One-cell embryos were microinjected with CRISPR/Cas9 ribonucleoprotein complexes targeting *cyp1b1* exon 1. Morphology of four crispant larvae with variable phenotypic manifestation was assessed microscopically at 144 hpf. **(WT)** Non injected wild type larvae were used as controls. **(Ctrl)** Control (no crRNA) microinjected embryos. White arrow: lower jaw underdevelopment. Red arrowhead: altered swim bladder development. Red dotted circle: wild type ocular periphery is indicated as a reference to show microphthalmia. Yellow arrowhead: pericardial edema. Scale bar = 250µm (A-D).

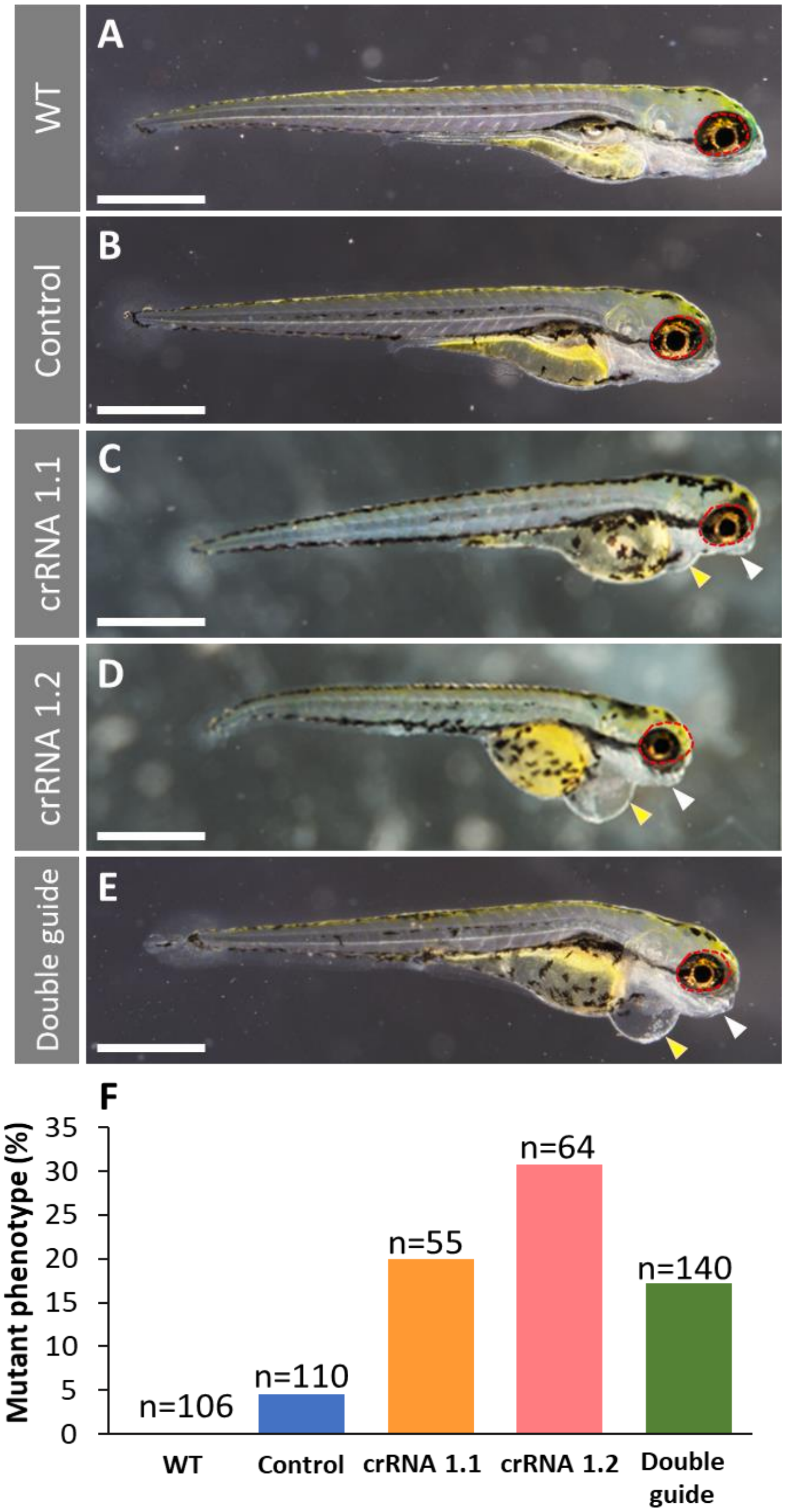

Figure S4. Phenotypic characterization of *cyp1b1* F0 crispants (96 hpf) obtained by independent or simultaneous microinjection of two single crRNA guides. One-cell embryos were microinjected with CRISPR/Cas9 ribonucleoprotein complexes with the indicated crRNAs targeting *cyp1b1* exon 1. (A) Crispants' morphology was assessed microscopically at 96 hpf. Non microinjected (WT) and control microinjected (control) larvae were used as controls. White arrow: lower jaw underdevelopment. Red dotted circle: wild type ocular periphery is indicated as a reference to show microphthalmia. Yellow arrowhead: pericardial edema. Scale bar= 500 µm. (D) Proportion of F0 mutant phenotypes, n indicate the number of embryos analyzed.

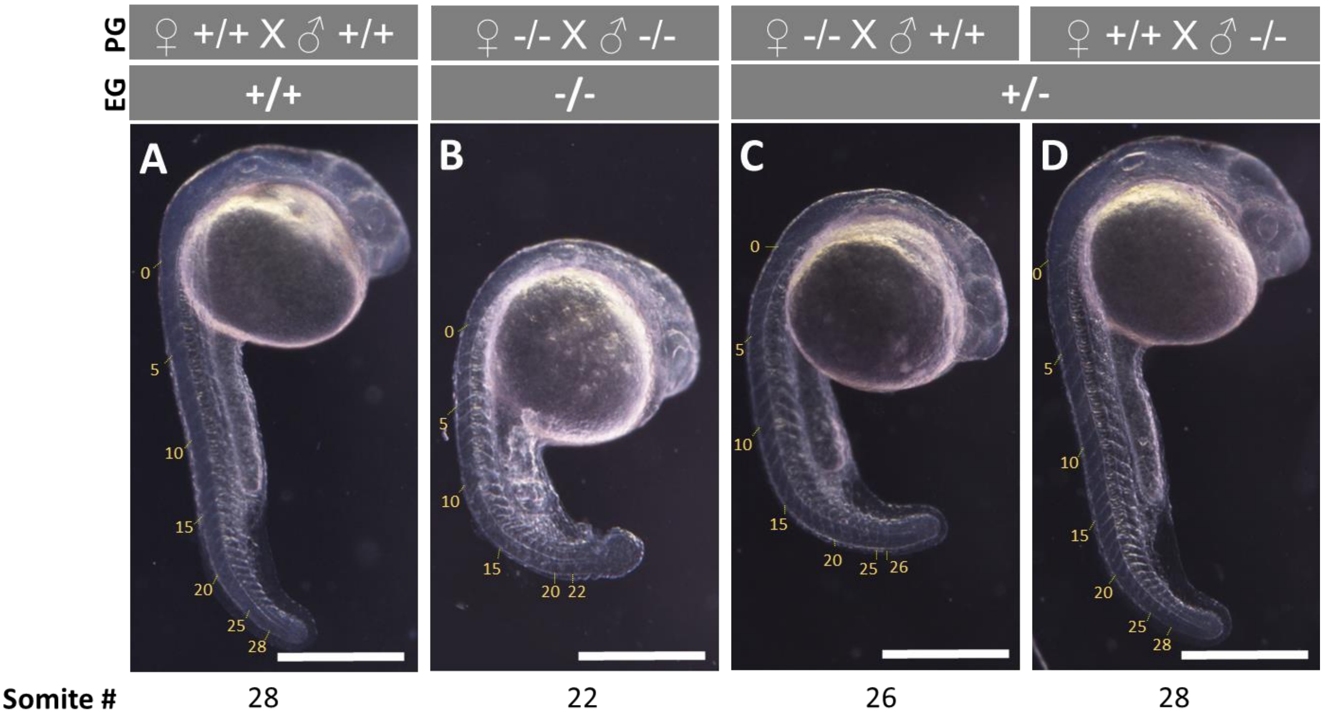

Figure S5. Somite development of *cyp1b1* +/+ , +/- and -/- zebrafish larvae (24 hpf) shown in figure 4. Images in A, B, C, D correspond to panels B, F, J, N in figure 4, respectively. EG: embryo genotype. PG: parental genotype. ♀: female. ♂: male. Yellow lines indicate somite number. All images are representative of the results observed in 10 embryos of each treatment. Scale bar = 500μm.

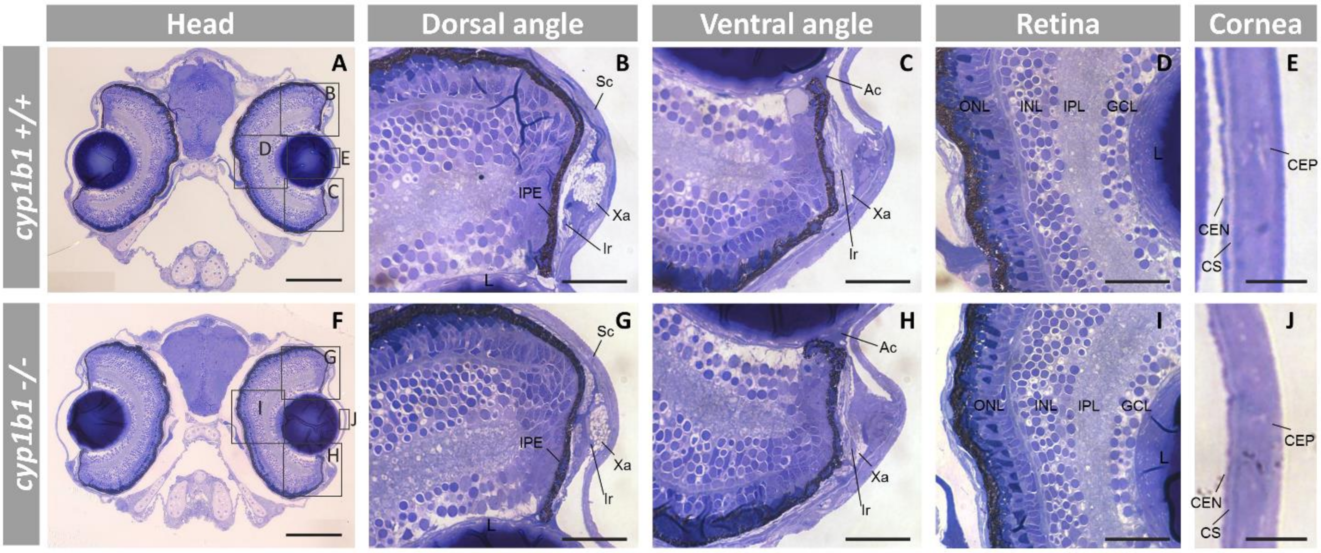

Figure S6. Histology of the established *cyp1b1*-KO zebrafish line (168hpf). The histological phenotypes were analyzed in F4 zebrafish obtained by inbreeding of young (<6 months) F3 siblings. (A-K) **S**emi-thin (500 nm) tissue sections were stained with Toluidine blue. The squares and rectangles indicate the areas of the images magnified in the indicated panels**.** No significant differences were observed between the eyes of wild type and *cyp1b1*-KO zebrafish siblings. Scale bar in A and F: 100 µm. Scale bar in B-D and G-I: 25 µm. Scale bar in E and J: 5 µm. Ac: Anterior chamber. CEN: Corneal Endothelium. CEP: Corneal Epithelium. CS: Corneal Stroma. GCL: Ganglion Cell Layer. IPE: Iris Pigmented Epithelium. Sc: Sclera. INL: Inner Nuclear Layer. IPL: Inner Plexiform Layer. Ir: Iridophores. L: Lens. ONL Outer Nuclear Layer. Xa: Xantophores. These images are representative of at least 10 histological sections of three different larvae of each genotype.

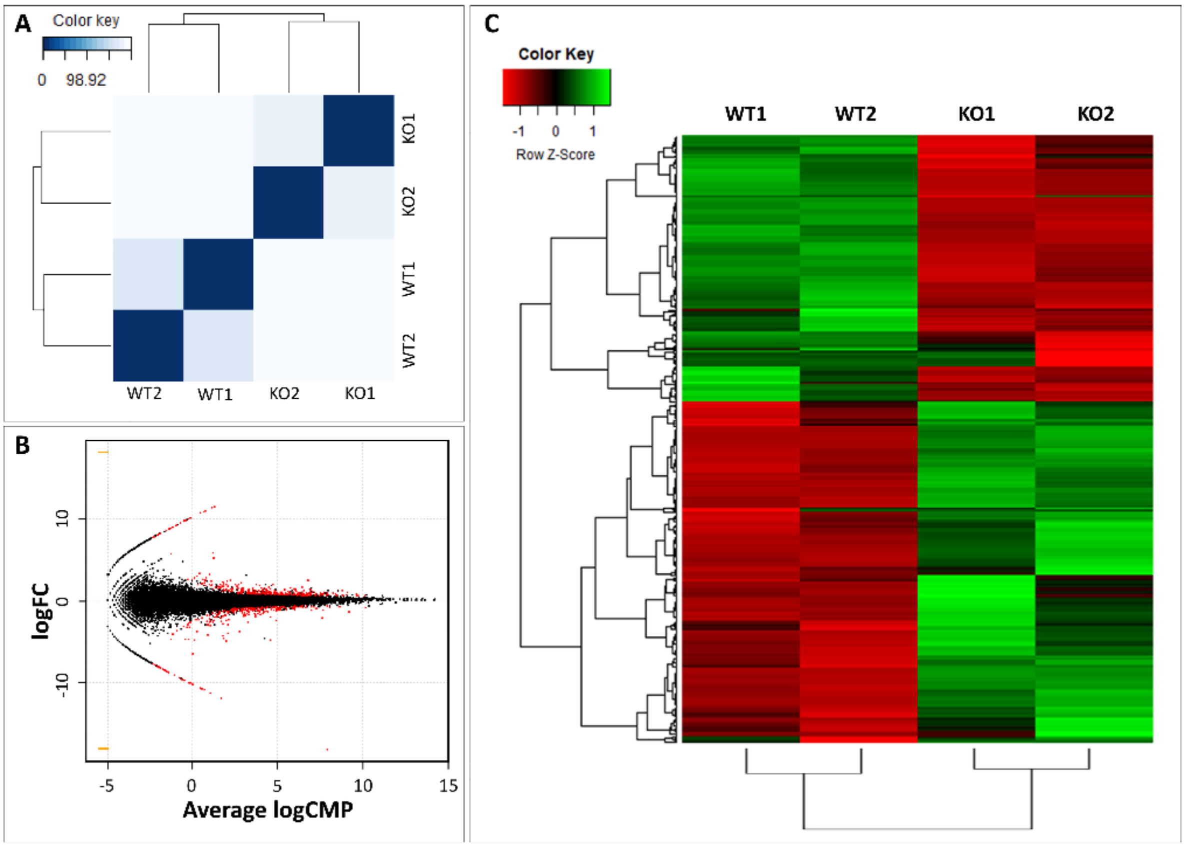

Figure S7. Differential expression analyses of RNA-seq data obtained from *cyp1b1* knockout and wild type zebrafish larvae (168 hpf). (A) Correlation matrix of all samples, showing the similarity between samples. Pearson's coefficient of sample's normalized value ($-1 \leq r \leq 1$) is used to evaluate similarity between samples. The closer to zero the greater the similarity. (B) MA plot of logFC and LogCPM of all tested genes (wild type vs. *cyp1b1* knockout). Dots above zero denote upregulated genes whereas red dots below zero indicate downregulated genes. Red dots correspond to significantly differentially expressed genes ($|\log_2(FC)| > 1$, p-value < 0.05). FC: fold change. CPM: counts per million. (C) Heatmap of hierarchical clustering showing significantly DEGs which are represented by red dots in B. Clustering was done using z-score for normalized values (log10 based, wild type vs. *cyp1b1* knockout). The color key indicates the intensity associated with normalized expression values: Red, green and black represent the highest, the lowest and no different expression levels, respectively.

Table S1. List of 451 DEGs of the *cyp1b1*-KO with an absolute fold change (FC) of at least 2, and a p-value <0.05 (185 up- and 266 down-regulated). Upper case gene names correspond to human orthologues of zebrafish unnamed genes.

Gene name	FC	P-Value	Function
ackr4a	2.1	5.2E-03	Atypical chemokine receptor 4
aco1	-2.9	1.6E-11	Aconitase 1
acot12	-2.9	2.0E-03	Acyl-CoA thioesterase 12
acta1b	-2.6	7.2E-17	Actin alpha 1, skeletal muscle
ADAMTS20 (LOC101886654)	-2.6	1.6E-02	A disintegrin and metalloproteinase with thrombospondin motifs 20-like
adcy1a	-2.1	4.1E-04	adenylate cyclase 1
ADGRL2 (LOC100535070)	2.8	4.6E-05	Adhesion G protein-coupled receptor L2-like
adra2b	2.3	2.4E-02	Alpha-2B adrenergic receptor-like
ADTRP (LOC100537455)	-2.5	7.3E-04	Androgen-dependent TFPI-regulating protein-like
aep1	-2.1	2.5E-03	Aerolysin-like protein
AGL (LOC101886684)	-2.6	1.6E-09	Glycogen debranching enzyme-like
ahrra	2.2	1.2E-02	Aryl-hydrocarbon receptor repressor
ak7a	2.6	2.2E-09	Adenylate kinase 7
ak8	2.8	1.6E-02	Adenylate kinase 8
alp3	-2.7	1.5E-02	Alkaline phosphatase
alpk3a	2	4.0E-08	Alpha kinase 3
amotl1	-2	1.3E-02	Angiomotin like 1
ampd3b	2.1	2.8E-07	Adenosine monophosphate deaminase 3
apoa4b.3	-2.9	1.6E-06	Apolipoprotein A4
apoea	-2.2	4.1E-05	Apolipoprotein E
aqp7	-2.1	3.3E-02	Aquaporin 7
aqp8a.2	-2.1	8.0E-03	Aquaporin 8
arf3b	-2.1	2.1E-03	ADP ribosylation factor 3
arsh	-2.2	4.0E-03	Arylsulfatase
asb2a.2	2.3	1.8E-02	Ankyrin repeat and SOCS box containing 2
asf1ba	-2.1	1.2E-03	Anti-silencing function 1B histone chaperone
BCAM (LOC100536698)	-2.3	3.5E-02	Basal cell adhesion molecule-like
BDP1 (LOC553492)	-2.2	1.6E-02	Transcription factor TFIIIB component B'' homolog
bsk146	-2.9	1.7E-12	Brain specific kinase 146
bub1bb	-2	1.1E-02	BUB1 mitotic checkpoint serine/threonine kinase B
C1QDC1L (si:dkeyp-110c7.4)	3.3	8.2E-11	C1q domain tumour necrosis factor-like domain superfamily
c6ast3	2.5	3.7E-05	Six-cysteine containing astacin protease 3
c6ast4	2.1	9.7E-04	Six-cysteine containing astacin protease 4
ca4c	2.2	3.8E-02	Carbonic anhydrase 4
calhm2	-2.2	8.0E-03	Calcium homeostasis modulator family member 2
calr3b	-2	5.6E-06	Calreticulin 3
CAPN1 (si:dkeyp-50d11.2)	-2.1	3.8E-05	Calpain 1
CARD8 (si:ch211-195h23.3)	2.4	3.9E-11	Caspase recruitment domain family member 8
casp7	3.2	2.1E-17	Caspase 7
casp8l2	-3.1	3.3E-02	Caspase 10
ccdc149a	3.1	1.5E-02	Coiled-coil domain containing 149
ccl19a.1	-2.3	2.0E-04	C-C motif chemokine ligand 19

Gene name	FC	P-Value	Function
ccn1l1	-5.6	8.0E-10	Cellular communication network factor 1, like 1
ccnd2b	-2.2	1.5E-02	Cyclin D2
CD58 (si:dkey-11f4.14)	-2.6	9.6E-06	CD58 molecule. Member of the immunoglobulin superfamily
cdc45	-2.1	4.1E-03	Cell division cycle 45
cdca8	-2.1	1.5E-03	Cell division cycle associated 8
cdk6	-2	5.9E-03	Cyclin dependent kinase 6
cel.2	2.5	2.8E-04	Carboxyl ester lipase
cep135	-3.3	5.7E-14	Centrosomal protein 135
cfd	2.4	7.3E-10	Complement factor D
ch25h	-3.1	1.3E-03	Cholesterol 25-hydroxylase
chia.3	-2.3	3.4E-03	Chitinase
CLDN23 (si:ch211-95j8.2)	2.3	1.1E-07	Claudin 23
clec19a	-2.5	4.3E-02	C-type lectin domain containing 19A
clip2	-2.1	2.9E-07	CAP-Gly domain containing linker protein 2.
cln8	-2.2	2.6E-02	CLN8 transmembrane ER and ERGIC protein
col8a1a	-2	3.6E-05	Collagen type VIII alpha 1 chain
cpeb4a	-2.3	7.4E-11	Cytoplasmic polyadenylation element binding protein 4
cpt2	-2.1	3.9E-06	Carnitine palmitoyltransferase 2
crb1	-2.2	7.6E-04	Crumbs cell polarity complex component 1
crybb1l3	-2.1	6.8E-03	Crystallin, beta B1, like 3
CSPG4 (si:dkey-193c22.2)	4.2	1.6E-02	Chondroitin sulfate proteoglycan 4-like
cspg4b	-2.1	1.6E-02	Chondroitin sulfate proteoglycan 4b
cspg4bb	2.9	6.3E-09	Chondroitin sulfate proteoglycan 4bb
cthl	-2.4	3.1E-06	Cystathionine gamma-lyase.
ctsd	2.2	1.2E-04	Cathepsin D
ctsll	4.9	4.5E-08	Cathepsin L
ctss1	2.1	4.4E-04	Cathepsin K
CXCL1 (LOC101883994)	-3.2	1.7E-02	Growth-regulated alpha protein-like
cxcl18b	-2.2	1.4E-03	Chemokine (C-X-C motif) ligand 18b
cyp11c1	-2.8	1.1E-04	cytochrome P450, family 11, subfamily C, polypeptide 1
cyp1b1	-2.3	2.7E-03	Cytochrome P450 family 1 subfamily B member 1
cyp24a1	2.7	3.3E-13	Cytochrome P450 family 24 subfamily A member 1
cyp4v8	-2.3	6.9E-04	Cytochrome P450 family 4 subfamily V member 2
cyp7a1	-2.5	9.5E-07	Cytochrome P450 family 7 subfamily A member 1
cyp8b1	-3.1	4.1E-05	Cytochrome P450 family 8 subfamily B member 1
dcdc2b	-3.1	9.1E-12	Doublecortin domain containing 2B
dcun1d4	-2.3	9.8E-11	Defective in cullin neddylation 1 domain containing 4
dhrs13l1	-2.1	1.9E-06	Dehydrogenase/reductase 13
dolk	-2.2	1.7E-02	Dolichol kinase
dsc2l	2.3	1.1E-09	Desmocollin 1-3
dsg2.1	2.1	3.1E-07	Desmoglein 2
duox	2.4	2.4E-02	Dual oxidases
dusp3b	2.4	3.6E-02	Dual specificity phosphatase 3
e2f7	-2	6.0E-04	E2F transcription factor 7
eepd1	2.1	4.4E-06	Endonuclease/exonuclease/phosphatase family domain containing 1
egr2a	-2	5.9E-06	Early growth response 2

Gene name	FC	P-Value	Function
elf3	-2.4	8.9E-08	E74 like ETS transcription factor 3
elovl8b	-3.8	4.0E-04	ELOVL fatty acid elongase 8b
enpp7.1	-3.1	3.3E-09	Ectonucleotide pyrophosphatase/phosphodiesterase 7
epgn	-2.4	4.4E-07	Epithelial mitogen
epsti1	2	3.5E-02	Epithelial stromal interaction 1
esr2b	2.2	1.3E-03	Estrogen receptor 2
f3b	-2.3	1.3E-07	Coagulation factor III, tissue factor
fabp11b	-2.1	3.0E-03	Fatty acid binding protein X
fam126a	2.7	9.4E-14	Family with sequence similarity 126 member A
FAM126B (LOC100536464)	2.1	1.9E-02	Family with sequence similarity 126 member B
fam159a	-3.6	3.4E-02	Family with sequence similarity 159 member A
fam167aa	-2.6	1.3E-02	Family with sequence similarity 167 member A
far1	-2	1.5E-07	Fatty acyl-CoA reductase 1
fbxo32	2	4.7E-10	F-box protein 32
FCRL5 (LOC570739)	2.5	5.0E-02	Fc receptor-like protein 5
fgf18a	-2.5	1.1E-02	Fibroblast growth factor 18
fgf4	-2	1.1E-03	Fibroblast growth factor 4
fhl3a	-2	7.5E-06	Four and a half LIM domains 3
FILIP1L (LOC101884302)	-2.2	2.9E-02	Filamin-A-interacting protein 1-like
fkbp5	2.1	6.9E-11	FKBP prolyl isomerase 5
FMO1 (LOC793236)	-3.8	1.5E-03	Flavin-containing monooxygenase
fosab	-2.2	4.9E-10	Fos proto-oncogene, AP-1 transcription factor subunit
fosl1a	-3.4	1.2E-08	FOS like 1, AP-1 transcription factor subunit
foxj2	2.3	2.4E-06	Forkhead box J2
fsta	2	1.1E-07	Follistatin
fthl29	3.4	2.3E-06	Ferritin, heavy polypeptide-like 29
fthl31	2.3	3.2E-09	Ferritin, heavy polypeptide-like 31
ftr02	2.2	3.1E-02	FinTRIM family member 2
ftr14l	6.5	4.9E-06	FinTRIM family member 14-like
furina	3.9	5.6E-19	Furin
fxyd7	-2	3.4E-08	FXYD Domain Containing Ion Transport Regulator 7
g2e3	-2.1	1.6E-03	G2/M-phase specific E3 ubiquitin protein ligase
gabpb2b	-2.4	1.5E-02	GA binding protein transcription factor subunit beta 2
gal3st2	-2.1	5.0E-03	Galactose-3-O-sulfotransferase 2
gbe1a	2.8	2.1E-03	1,4-alpha-glucan branching enzyme 1
gc2	-2.2	3.5E-02	Guanylate cyclase 2F, retinal
gcga	-2.1	4.5E-03	Glucagon
gdf2	2.5	1.1E-02	Growth differentiation factor 2
GIMAP4 (LOC101884484)	2.5	4.1E-02	GTPase IMAP family member 4-like
gkup	2.8	3.8E-02	Glucuronokinase
gng10	2	3.3E-02	G protein subunit gamma 10
gnrh3	-2	3.6E-02	Gonadotropin releasing hormone 1
gnrhr2	3.2	3.9E-02	Gonadotropin releasing hormone receptor 2
GOLGA6L22 (LOC103909796)	-2.8	3.3E-03	Golgin subfamily A member 6-like protein 2
grb2a	-2.2	2.6E-05	Growth factor receptor bound protein 2
grhl3	3.2	7.3E-07	Grainyhead like transcription factor 3
grm1b	-2.1	2.3E-02	Glutamate metabotropic receptor 1
grxcr1	2.4	2.7E-05	Glutaredoxin And Cysteine Rich Domain Containing 1

Gene name	FC	P-Value	Function
gstt2	-2.5	1.6E-04	Glutathione S-transferase theta 2
hephl1b	3.8	1.7E-02	Hephaestin like 1
heyl	-2.3	3.2E-03	Hes related family bHLH transcription factor with YRPW motif like
hhla2a.2	3.1	3.5E-05	HERV-H LTR-associating 2
hnf4a	-3.3	3.6E-02	Hepatocyte nuclear factor 4 alpha
hsd11b2	2.1	8.2E-08	Hydroxysteroid 11-beta dehydrogenase 2
ido1	-4.5	1.5E-03	Indoleamine 2,3-dioxygenase 1
igfbp1b	4	2.6E-11	Insulin like growth factor binding protein 1
igfbp2b	-2.3	1.8E-02	Insulin like growth factor binding protein 2
il12bb	3.3	2.6E-02	Interleukin 12B
il1b	-2.8	1.8E-02	Interleukin 1 beta
INHBB (LOC103909000)	-2.6	3.9E-02	Inhibin beta B chain-like
insc	2.7	1.9E-03	Inscuteable Spindle Orientation Adaptor Protein
insl3	2.1	1.1E-02	Insulin like 3
iqcc	-3.1	1.2E-05	IQ motif containing C
itgae.1	3.8	1.3E-04	Integrin subunit alpha E
itgae.2	-2.6	1.9E-02	Integrin subunit alpha E
itgb4	2	6.9E-08	Integrin subunit beta 4
jac1	-4.1	1.6E-02	Jacalin 1 (lectin)
jac8	2.8	2.8E-02	Jacalin 8 (lectin)
jdp2b	-3.1	1.8E-15	Jun dimerization protein 2
jmjd8	-2.4	3.7E-02	Jumonji domain containing 8
junba	-2.1	2.4E-10	JunB proto-oncogene, AP-1 transcription factor subunit
junbb	-2.2	5.9E-10	JunB proto-oncogene, AP-1 transcription factor subunit
KBTBD13 (LOC101886608)	-2.1	2.6E-02	Kelch repeat and BTB domain-containing protein 13-like
kbtbd7	-2.3	1.4E-03	Kelch repeat and BTB domain containing 7
kdelc1	-2.6	3.0E-05	protein O-glucosyltransferase 2
khnyn	-2	9.0E-04	KH and NYN domain containing
KIAA0753 (si:dkey/243i1.1)	-2.6	1.6E-11	Protein moonraker. Involved in centriole duplication
klf10	-4.8	5.7E-03	Kruppel like factor 10
KLHL33 (si:ch211-63p21.8)	2.3	7.9E-04	Kelch like family member 33
lamc2	2.8	3.8E-05	Laminin subunit gamma 2
lmln	-2.2	1.7E-02	Leishmanolysin like peptidase
lnx1	2.3	3.6E-13	Ligand of numb-protein X 1
LOC100005008	2.6	3.0E-04	ARAP1-arf-GAP with Rho-GAP domain, ANK repeat and PH domain-containing protein 1-like
LOC100005923	-2.4	1.3E-02	Gastrula zinc finger protein XlCGF49.1-like
LOC100006428	2.2	9.7E-07	Ferritin
LOC100007488	-2	3.9E-02	MFAP4 -microfibril-associated glycoprotein 4-like
LOC100147849	2.8	4.6E-02	Mucin
LOC100149234	3.8	3.7E-03	GVIN1-interferon-induced very large GTPase 1-like
LOC100330257	-2.5	7.5E-04	RAB39B-ras-related protein Rab-39A-like
LOC100334202	-3.1	3.5E-08	NLRP3-NACHT, LRR and PYD domains-containing protein 3-like.
LOC100535428	-4.4	2.7E-10	NACHT, LRR and PYD domains-containing protein 3-like
LOC101882592	3.1	2.7E-02	Predicted to have G protein-coupled receptor activity

Gene name	FC	P-Value	Function
LOC101885212	-3.1	1.8E-02	NLRP3-NACHT, LRR and PYD domains-containing protein 3-like
LOC558816	5.7	3.1E-07	Ferritin, middle subunit-like
LOC559107	-2.4	7.8E-04	Lactase-phlorizin hydrolase
loxl4	5.2	2.0E-14	Lysyl oxidase like 4
loxl5b	-2.1	7.3E-03	Lysyl oxidase like 5
lratb.2	-2.2	4.1E-06	Lecithin retinol acyltransferase b, tandem duplicate 2
lrch2	-2.3	2.8E-05	Leucine rich repeats and calponin homology domain containing 2
lrrc10b	-2.1	4.4E-02	Leucine rich repeat containing 10
ltb4r2b	2.7	4.2E-02	Leukotriene B4 receptor
lyz	-2.7	3.6E-06	Lysozyme like 1
maff	2	2.3E-08	MAF bZIP transcription factor F
mbtps1	2	1.2E-06	Membrane-bound transcription factor peptidase, site 1
me1	-4.7	1.8E-03	NADP-dependent malic enzyme-like
megf6b	-2.2	1.4E-03	Multiple EGF like domains 6
mfsd2aa	-5.4	2.3E-12	Major facilitator superfamily domain containing 2A
mgat3a	-2.9	8.9E-03	β-1,4-mannosyl-glycoprotein 4-β-N-acetylglucosaminyltransferase
mhc1ula	-2.9	8.2E-04	Major histocompatibility complex, class I-related
mkks	-2.2	4.5E-03	McKusick-Kaufman syndrome
mlphb	-2.4	3.5E-05	Melanophilin
mmp11b	-2.9	5.6E-03	Matrix metallopeptidase 11
mmp28	2.1	1.8E-02	Matrix metallopeptidase 28
mns1	2.2	6.9E-04	Meiosis specific nuclear structural 1
mtmr12	3	1.9E-13	Myotubularin related protein 12
MUL1 (LOC402879)	2.6	1.5E-06	Mitochondrial ubiquitin ligase activator of nfkb 1-A
mybpha	-2.3	1.5E-05	Myosin binding protein H
ncam2	-2.1	3.5E-08	Neural cell adhesion molecule 2
ncapg2	-2.2	7.2E-03	Non-SMC condensin II complex subunit G2
ndrg4	-4.2	7.9E-27	NDRG family member 4
nfr	3.2	3.2E-02	Notochord formation related gene
nipsnap1	-2.3	2.9E-02	nipsnap homolog 1
nkx6.3	2.4	3.5E-02	NK6 homeobox 3
nlrb5	2.7	3.7E-02	NOD-like receptor family B, member 5
nlrp12	-7.6	1.4E-19	NACHT, LRR and PYD domains-containing protein 12-like
notum2	-2.1	1.8E-02	Notum pectinacetylesterase 2
npffl	-2.6	2.9E-04	Neuropeptide FF-amide peptide precursor
npsn	-2.2	2.2E-05	Astacin like metalloendopeptidase
npy2r	2.6	5.0E-02	Neuropeptide Y receptor Y2
nr0b2a	-2.4	1.7E-02	Nuclear receptor subfamily 0 group B member 2.
nr4a3	2.5	2.7E-14	Nuclear receptor subfamily 4 group A member 3
nsl1	-2.3	3.2E-02	NSL1 component of MIS12 kinetochore complex
nudt3b	-2.1	8.1E-04	Nudix (nucleoside diphosphate linked moiety X) hydrolase 3
numa1	-2.1	2.7E-05	Nuclear mitotic apparatus protein 1
olfml2ba	-3.5	2.6E-12	Olfactomedin like 2B
ompb	-3.2	1.6E-02	Olfactory marker protein
opn4xa	-2.5	4.8E-02	Opsin 4xa

Gene name	FC	P-Value	Function
or111-11	-4.1	8.3E-03	Odorant receptor, family D, subfamily 111, member 11
or115-11	-3.3	2.0E-03	Odorant receptor, family D, subfamily 115, member 11
osbpl3a	2.4	4.1E-05	Oxysterol binding protein like 3
paqr3b	4.1	5.5E-05	Progestin and adipoQ receptor family member 3
PCBP3 (zgc:162999)	-2.6	2.9E-02	Poly(rC)-binding protein 3-like
pcdh1g2	2.1	5.0E-07	Protocadherin 1 gamma 2
pcdh1g26	3	3.6E-12	Protocadherin 1 gamma 26
pcdh1g3	-2.1	1.1E-03	Protocadherin 1 gamma 3
pcdh1g30	-4	5.0E-11	Protocadherin 1 gamma 30
pcdh1gb9	-5.9	7.2E-13	Protocadherin 1 gamma b 9
pcyt1bb	-2.3	4.4E-02	Phosphate cytidylyltransferase 1, choline, beta
pde9al	2.4	2.2E-02	Phosphodiesterase 9A
pdxka	-2.4	2.7E-07	Pyridoxal kinase
pdzd3b	-2.9	4.8E-03	PDZ domain containing 3
pgpep1	-2.2	9.8E-03	Pyroglutamyl-peptidase I
pigr	-2.6	2.2E-04	Polymeric immunoglobulin receptor
pik3ap1	-2	3.6E-02	Phosphoinositide-3-kinase adaptor protein 1
PIM2 (LOC103909000)	4	3.3E-03	Serine/threonine-protein kinase pim-2-like
pimr130	-3.2	4.2E-02	Pim proto-oncogene, serine/threonine kinase, related 130
pimr197	2.6	2.8E-02	Pim proto-oncogene, serine/threonine kinase, related 197
pkd1l1	3.4	2.0E-02	Polycystin 1 like 1
pkhd1l1	2.2	8.3E-07	PKHD1 like 1
pld2	-6	1.6E-25	Phospholipase D2
plekhs1	-3.3	2.8E-02	Pleckstrin homology domain containing S1
plin2	2.8	8.6E-09	Perilipin 2
plpp4	-3.1	2.2E-03	Phospholipid phosphatase 4
pltp	2	1.0E-06	phospholipid transfer protein
pnpla1	-2.3	5.0E-02	Patatin like phospholipase domain containing 1
ponzr6	2.3	1.4E-02	Plac8 onzin-related protein 6
postna	-2.4	3.5E-08	Periostin
ppifa	-2.2	1.7E-08	Peptidylprolyl isomerase F
ppm1f	-3.2	4.1E-02	Protein phosphatase, Mg2+/Mn2+ dependent 1F
ppp1r27b	2	6.8E-04	Protein phosphatase 1 regulatory subunit 27
ppp1r3b	-3.1	2.0E-02	Protein phosphatase 1 regulatory subunit 3B
prg4b	2.1	4.6E-06	Proteoglycan 4
prp	-2.1	5.1E-05	Prion protein. Glycosylphosphatidylinositol-anchored glycoprotein
pskh1	-4.2	3.1E-05	Protein serine kinase H1
psmb7	2.1	4.5E-10	Proteasome 20S subunit beta 7
psmc1b	2	3.6E-09	Proteasome 26S subunit, ATPase 1
psme1	2.1	4.3E-04	Proteasome activator subunit 1
ptpn20	-2.3	1.8E-10	Protein tyrosine phosphatase non-receptor type 20
ptrh2	2.3	3.9E-07	Peptidyl-tRNA hydrolase 2
pycr1a	2.1	1.0E-03	Pyrroline-5-carboxylate reductase 1
qkia	2.2	4.7E-11	QKI, KH domain containing RNA binding
rab42b	2.4	1.5E-02	Member RAS oncogene family
rab43	3.8	2.9E-09	Member RAS oncogene family

Gene name	FC	P-Value	Function
rac1	-3.2	6.9E-04	ras-related C3 botulinum toxin substrate 1-like
racgap1	-2.3	5.1E-03	Rac GTPase activating protein 1
rbm25a	2.5	1.4E-12	RNA binding motif protein 25
rbp1	-3.4	8.4E-03	Retinol binding protein 1
rbp2b	-2.2	3.8E-06	Retinol binding protein 2
rdh12l	2.3	2.3E-04	Retinol dehydrogenase 12 like
rdh20	-2.4	6.1E-04	Retinol dehydrogenase 20
relb	-2.3	7.6E-06	RELB proto-oncogene, NF-kB subunit
RFTN1 (LOC101885083)	-3.1	1.9E-02	Raftlin, Lipid Raft Linker 1
rgs13	-2.1	3.4E-03	Regulator of G protein signaling 13
rh50	-2.7	4.2E-03	Rh50-like protein
RNF14 (LOC100537178)	-2.1	3.3E-02	The protein encoded by this gene contains a RING zinc finger
rnf150	-2.2	4.9E-02	Ring finger protein 150
RNF39 (si:dkey/219e21.2)	-2.7	2.3E-06	Ring finger protein 39
rps6kb1a	2	3.4E-04	Ribosomal protein S6 kinase B1
rrh	-3.1	5.4E-06	Retinal pigment epithelium-derived rhodopsin homolog
sb:cb288	-2.2	9.0E-04	Uncharacterized zebrafish gene
sc5d	-2.2	4.0E-02	Sterol-C5-desaturase
scarf2	-2.8	9.1E-04	Scavenger receptor class F member 2
scn4bb	-2.1	3.2E-02	Sodium voltage-gated channel beta subunit 4
sele	-3.2	1.6E-06	Selectin E
sell	-2.7	7.1E-03	Selenoprotein L
shpk	2.1	3.3E-02	Sedoheptulokinase
si:busm1-105l16.2	3.8	1.3E-09	Predicted to have microtubule binding activity
si:cabz01036006.1	5.1	1.3E-08	Predicted to have transmembrane signaling receptor activity
si:ch1073-13h15.3	-3	1.2E-04	Predicted to have GDP-dissociation inhibitor, GTPase activator and oxidoreductase activity
si:ch1073-143l10.2	-2.7	1.7E-02	Predicted to be involved in autophagosome
si:ch1073-147h9.1	2.1	1.6E-04	Uncharacterized zebrafish gene
si:ch211-207e14.4	-2.9	2.7E-07	Predicted to have interleukin-17 receptor activity
si:ch211-217g15.3	-3.6	5.2E-07	Uncharacterized zebrafish gene
si:ch211-220d9.3	-2.4	7.8E-06	Enkurin domain containing 1
si:ch211-220i18.4	2.4	3.0E-04	Predicted to have MAP kinase activity
si:ch211-241d24.1	2.9	1.4E-02	Solo transcritos no codificantes descritos
si:ch211-241e1.3	2.8	2.1E-08	Predicted to have signaling receptor binding activity
si:ch211-243p7.3	2.1	3.4E-04	Uncharacterized zebrafish gene
si:ch211-25d12.7	3.5	8.9E-03	Src like adaptor 2
si:ch211-261n11.8	3	1.2E-09	TNF receptor superfamily member 14
si:ch211-107n13.1	-3.5	4.0E-05	Family with sequence similarity 110 member D
si:ch211-133h13.1	-2.8	2.2E-02	Predicted to be involved in intracellular signal transduction
si:ch211-133l11.10	-2.6	4.4E-02	Ras homolog family member U
si:ch211-14c7.2	5.6	3.0E-32	Uncharacterized zebrafish gene
si:ch211-156l18.8	-2	1.1E-02	G-protein coupled receptor 158
si:ch211-157c3.4	2.5	4.2E-07	Predicted to have metal ion binding activity
si:ch211-176g13.8	-6.1	3.9E-05	Predicted to have lipid binding activity
si:ch211-178n15.1	-2.6	1.3E-02	Domains Protein Largen/Inhibitory synaptic factor 1

Gene name	FC	P-Value	Function
si:ch211-199m9.3	-3.2	3.0E-03	Unmapped lincRNA
si:ch211-66i8.3	-3.4	6.3E-04	Unmapped lincRNA
si:ch211-69l10.2	2.3	7.6E-03	Encodes a long, intervening non-coding RNA
si:ch73-226l13.2	-2.1	2.4E-02	Predicted to have cytokine activity
si:ch73-263o4.4	-2.3	3.0E-02	Serine racemase
si:ch73-334d15.1	-2.1	2.1E-02	Predicted to have UDP-glycosyltransferase activity
si:ch73-335l21.4	-2.7	2.8E-12	Predicted to have metal ion binding and transferase activity
si:ch73-18b11.2	-2.5	1.1E-02	Uncharacterized zebrafish gene
si:dkey-208k4.3	-3	8.4E-03	Uncharacterized zebrafish gene
si:dkey-22i16.9	-3.9	1.4E-07	Predicted to be involved in cell surface receptor signaling
si:dkey-23n7.10	2.3	2.4E-06	Predicted to be involved in proteasome-mediated ubiquitin-dependent protein catabolic process
si:dkey-266f7.9	2.1	5.3E-03	Predicted to have phosphoric diester hydrolase activity
si:dkey-10h3.1	7.2	6.7E-10	Sialoadhesin-like
si:dkey-111e8.5	3	4.2E-02	Metal ion binding and ubiquitin-protein transferase activity
si:dkey-190g11.3	2.4	4.5E-02	Predicted to have protein-lysine N-methyltransferase activity
si:dkey-192d15.2	3	1.6E-02	Apolipoprotein
si:dkey-1k23.3	3	1.9E-02	Alpha crystallin/Hsp20 domain
si:dkey-56d12.4	6	5.3E-05	Uncharacterized zebrafish gene
si:dkey-58f10.6	3.1	8.1E-03	Predicted to have GTP binding activity
si:dkey-61o18.2	-2.7	2.2E-02	Unmapped lincRNA
si:dkey-74i1.3	2.1	4.1E-02	Unmapped lincRNA
si:dkey-76k16.6	-3.6	5.1E-06	Predicted to have N-acyltransferase activity
si:dkey-7c18.24	-2.5	3.4E-07	Translation iniciation factor IL-2
si:dkey-9c18.3	2.1	7.8E-03	B cell receptor CD22 / sialoadhesin
si:dkey-9i23.15	-2	2.6E-04	Transmembrane protein 109
si:dkeyp-34c12.1	2.3	2.1E-04	Proteoglycan
si:rp71-45g20.11	-2.4	2.4E-02	Predicted to have ubiquitin protein ligase activity and zinc ion binding activity
si:rp71-77l1.1	2.1	5.2E-03	Dispanin subfamily A member 2b-like
si:zfos-2326c3.2	-2.2	2.6E-06	Misshapen-like kinase 1
sipa1l3	-2.2	1.4E-06	Signal induced proliferation associated 1 like 3
slc13a5b	-2.8	4.4E-02	Solute carrier family 13 member 5
slc15a1a	-2	5.6E-03	Solute carrier family 15 member 1
slc15a1b	-2	8.5E-05	Solute carrier family 15 member 1
slc25a15b	2	1.0E-05	Solute carrier family 25 member 15
slc25a47a	2.5	9.3E-10	Solute carrier family 25 member 47
slc30a1b	-3	2.1E-02	Solute carrier family 30 member 1
slc34a2a	-2.7	2.3E-04	Solute carrier family 34 member 2
slc43a1a	2.1	1.8E-04	Solute carrier family 43 member 1
slc47a3	-2.7	2.5E-04	Solute carrier family 47 member 3
slx4ip	-3.8	1.9E-07	SLX4 interacting protein
smcr8b	2	9.2E-04	SMCR8-C9orf72 complex subunit
SMIM18 (si:dkey-92j12.6)	2.3	2.9E-08	Small integral membrane protein 18
smpd5	2	1.4E-06	Sphingomyelin phosphodiesterase 5 (pseudogene)
socs3b	-2.1	1.3E-08	Suppressor of cytokine signaling 3

Gene name	FC	P-Value	Function
soul5l	-3.1	2.1E-02	Heme-binding protein soul5, like
SPACA5 (LOC100536671)	2.1	2.0E-02	Sperm acrosome-associated protein 5-like
spata6l	2.6	6.2E-07	Spermatogenesis associated 6 like
sptssb	3.9	3.4E-08	Serine palmitoyltransferase small subunit B
src	-2.4	3.7E-06	SRC proto-oncogene, non-receptor tyrosine kinase
srd5a2b	-4.2	3.3E-03	Steroid 5 alpha-reductase 2
SSC4D (LOC100331436)	2.4	8.8E-03	Scavenger Receptor Cysteine Rich Family Member With 4 Domains
st3gal7	-2.6	2.0E-04	ST3 beta-galactoside alpha-2,3-sialyltransferase 7
stoml3b	-2.2	2.6E-05	Stomatin like 3
stx11a	2.5	2.4E-02	Syntaxin 11
swap70b	2.1	2.0E-04	Switching B cell complex subunit SWAP70
syt18b	-2	5.4E-04	Synaptotagmin XVIIIb
tas1r1	3.1	4.2E-02	Taste 1 receptor member 1
tcaf1	-2	1.5E-02	TRPM8 channel associated factor 1
tekt1	2	4.2E-05	Tektin 1
tekt3	2.3	9.2E-03	Tektin 3
thap1	2.5	4.2E-08	THAP domain containing 1
THBS1 (LOC100535166)	-2.3	6.6E-04	Thrombospondin-1-like
thfp5	-5	1.2E-13	Three-finger protein 5
tk1	-2	6.5E-04	Thymidine kinase 1
tmc1	2.8	3.2E-04	Transmembrane channel like 1
tmc2a	4.7	4.3E-03	Transmembrane channel like 2
tmem150ab	-2.5	1.3E-05	Transmembrane protein 150A
tmem163a	-3.7	5.2E-13	Transmembrane protein 163
tmem205	-2.1	2.6E-04	Transmembrane protein 205
tmem37	-2.5	1.5E-03	Transmembrane protein 37
tmem86b	-2.2	7.4E-04	Transmembrane protein 86B
tmprss13b	2.2	3.0E-05	Transmembrane serine protease 13
tmtops3b	-2.1	2.5E-02	Teleost multiple tissue opsin 3b
TNFRSF14 (si:ch211/261n11.8)	3.8	1.2E-09	TNF receptor superfamily member 14
tnfsf13b	-23.8	4.2E-30	TNF superfamily member 13b
tnni4b.1	2.8	2.3E-02	Troponin I4b, tandem duplicate 1
tprg1	-2	5.1E-04	Tumor protein p63 regulated 1
trim109	2.3	9.2E-07	Tripartite motif containing
trim63a	2.4	8.8E-15	Tripartite motif containing 63
tspan14	2.6	5.9E-10	Tetraspanin 14
ttc23	2.4	1.3E-02	Tetratricopeptide repeat domain 23
ttc7a	-2.2	1.8E-05	Tetratricopeptide repeat domain 7A
ttpal	-3	9.9E-12	Alpha tocopherol transfer protein like
txnipb	-2	3.9E-08	Thioredoxin interacting protein
txnl1	3.1	2.6E-15	Thioredoxin like 1
txnrd2.2	-3.5	1.8E-08	Thioredoxin reductase 2
tyms	-2	9.1E-04	Thymidylate synthetase
tyr	-2.6	2.2E-03	Tyrosinase.
tyro3	-2.3	4.5E-04	TYRO3 protein tyrosine kinase
ubb	2	1.8E-09	Ubiquitin C
ubl7b	2.2	1.0E-10	Ubiquitin like 7

Gene name	FC	P-Value	Function
ubxn10	2.2	4.1E-02	UBX domain protein 10
ugp2b	2.1	1.0E-08	UDP-glucose pyrophosphorylase 2
ugt1ab	-4.5	3.6E-06	UDP glucuronosyltransferase family 1 member A1
ugt2a2	6.3	5.2E-05	UDP glucuronosyltransferase 2 family, polypeptide A2
ugt5b3	-2.1	1.3E-02	UDP glucuronosyltransferase 5 family, polypeptide B3
utp23	2.1	4.9E-08	UTP23 small subunit processome component
vti1b	-2.1	1.1E-05	Vesicle transport through interaction with t-SNAREs 1B
vwa10.1	-3	1.1E-02	Von Willebrand factor A domain containing 7
wasf3b	-5.3	6.9E-36	WASP family member 3
wdr35	2.1	3.0E-08	WD repeat domain 35
wdr66	-4	1.1E-02	Cilia and flagella associated protein 251
wnt9b	-2.5	3.6E-05	Wnt family member 9B
wu:fd06e11	-2.1	3.5E-02	gastrula zinc finger protein XlCGF49
xirp1	2.5	2.4E-10	xin actin binding repeat containing 1
ydjc	-2.3	9.5E-05	YdjC chitooligosaccharide deacetylase homolog
ythdf3	2	1.1E-05	YTH N6-methyladenosine RNA binding protein 3
zbtb5	-2.3	9.7E-06	zinc finger and BTB domain containing 5
zgc:100868	2.9	1.7E-08	Serine protease 27
zgc:100920	2.7	5.2E-05	Sodium/potassium transporting ATPase interacting 4
zgc:101785	-5.4	3.7E-08	Annexin
zgc:103601	-3.1	4.9E-02	Arylamine N-acetyltransferase
zgc:109982	-2.1	4.8E-02	Retinol dehydrogenase 8
zgc:112160	2.1	5.5E-06	Chymotrypsinogen B2
zgc:114046	4.2	6.0E-04	Histone H2B
zgc:152945	-2.8	1.9E-03	Hemopexin
zgc:153431	-3	1.5E-03	CSC1-like protein 2
zgc:153759	3	3.6E-05	TNF receptor superfamily member 14
zgc:154142	-2.1	2.0E-05	Predicted to have serine-type endopeptidase activity
zgc:162999	2	8.1E-03	PCBP3 Poly(rC)-binding protein 3
zgc:172122	3.5	8.2E-06	CD200 molecule
zgc:174379	2.5	7.0E-03	Saccharopine dehydrogenase
zgc:174653	2.1	4.1E-02	Predicted to have DNA-binding transcription factor activity
zgc:198329	-8	1.0E-05	Carcinoembryonic antigen-related cell adhesion molecule 8
zgc:64201	2.1	4.6E-03	Prolyl-tRNA synthetase associated domain containing 1
zgc:86764	-2.3	6.5E-04	Predicted to have microtubule binding activity
zgc:92137	-2.9	1.1E-05	Predicted to have alpha-amylase activity
zgc:92745	3.4	3.7E-05	Chymotrypsin like elastase 1
znf750	4.1	4.3E-07	Zinc finger protein 750
znhit3	2.1	1.4E-04	Zinc finger HIT-type containing 3
Zp3	2.6	1.9E-03	Zona pellucida glycoprotein 3

Table S2. Biological process analysis of 451 DEGs in the *cyp1b1*-KO using the DAVID bioinformatic tool. Count indicates the number of genes included in each pathway.

Biological Process Term	Count	P-Value	Genes
Response to lipopolysaccharide	8	<0.0001	*cxcl18b, zgc:153759, il1b, junba, junbb, loc101883994, si:ch211-261n11.7, si:ch211-261n11.8*
Cell adhesion	17	<0.0001	*pcdh1g3, lmln, cyr61l1, dsg2.1, itgae.2, pcdh1g2, itgb4, src, pcdh1g26, dsc2l, sele, postna, pcdh1gb9, si:ch211-241e1.3, tyro3, ncam2, pcdh1g30*
Proteolysis	22	<0.0001	*lmln, cfd, casp8l2, mbtps1, tmprss13b, zgc:154142, c6ast3, c6ast4, zgc:92745, si:dkeyp-50d11.2,zgc:100868, psmb7, zgc:112160, casp7, ctsll, mmp11b, ctss1, mmp28, npsn, pgpep1, ctsd, furina*
Inflammatory response	10	0.001	*cxcl18b, ltb4r2b, elf3, zgc:153759, il1b, loc101883994, ccl19a.1, si:ch211-261n11.7, relb, si:ch211-261n11.8*
Regulation of cell proliferation	9	0.002	*cxcl18b, cdk6, zgc:153759, src,junba, junbb, loc101883994, si:ch211-261n11.7, si:ch211-261n11.8*
Neutrophil chemotaxis	5	0.004	*cxcl18b, il1b, duox, loc101883994, ccl19a.1*
Neutrophil activation	3	0.005	*cxcl18b, il1b, loc101883994*
Oxidation-reduction process	22	0.006	*hephl1b, pycr1a, loc793236, txnl1, srd5a2b, si:ch1073-179p4.3, loxl4, zgc:174379, si:ch1073-13h15.3, tyr, cyp7a1, cyp8b1, ch25h, cyp24a1, loxl5b, cyp11c1, sc5d, cyp1b1, duox, cyp4v8, zgc:109982, ido1*
Lipid metabolic process	10	0.009	*mbtps1, ch25h, smpd5, si:dkey-266f7.9, cpt2, elovl8b, srd5a2b,sptssb, pltp, cel.2*
Response to cytokine	3	0.014	*junba, junbb, relb*
Intracellular sequestering of iron ion	3	0.017	*loc558816, zgc:194125, zgc:109934*
Response to bacterium	4	0.020	*il1b, duox, ctsd,furina*
Iron ion transport	3	0.027	*loc558816, zgc:194125, zgc:109934*
Regulation of transcription from RNA polymerase II promoter	11	0.028	*fosl1a, nkx6.3, fosab, jdp2b, elf3,maff, junba, junbb, grhl3, ahrra, relb*
Lipid transport	5	0.042	*apoea, mfsd2aa, si:dkey-192d15.2, osbpl3a, apoa4b.3*
Homophilic cell adhesion via plasma membrane adhesión molecules	8	0.042	*pcdh1g3, dsg2.1, pcdh1g2, pcdh1gb9, pcdh1g26, zgc:172122, dsc2l, pcdh1g30*
Immune response	11	0.048	*cxcl18b, zgc:153759, il1b, ctss1, si:dkey-11f4.14, loc101883994, ccl19a.1, si:ch211-261n11.7, prg4b, tnfsf13b, si:ch211-261n11.8*

Table S3. Molecular Function analysis of 451 DEGs in the *cyp1b1*-KO using the DAVID bioinformatic tool. Count indicates the number of genes included in each pathway.

Molecular Function Term	Count	P-Value	Genes
Peptidase activity	21	<0.0001	*cfd, casp8l2, mbtps1, tmprss13b, zgc:154142, c6ast3, c6ast4, zgc:92745, si:dkeyp-50d11.2,zgc:100868, psmb7, zgc:112160, casp7, ctsll, mmp11b, ctss1, mmp28,npsn, pgpep1,ctsd, furina*
Iron ion binding	11	0.002	*loc558816, zgc:194125, ch25h, cyp24a1, zgc:109934, cyp11c1, sc5d, cyp1b1, cyp4v8, cyp7a1, cyp8b1*
Lipid binding	8	0.003	*rbp2b, apoea, rbp1, acot12, pltp, si:dkey-192d15.2, apoa4b.3, fabp11b*
Oxidoreductase activity	20	0.004	*hephl1b, pycr1a, si:ch1073-179p4.3, dhrs13l1, zgc:174379, si:ch1073-13h15.3, tyr, cyp7a1, cyp8b1, hsd11b2, ch25h, cyp24a1, rdh20, cyp11c1, sc5d, cyp1b1, duox, cyp4v8, zgc:109982, ido1*
Hydrolase activity	30	0.005	*cfd, c6ast3, c6ast4, ugt1ab, zgc:92745, ppm1f, alp3, zgc:100868, psmb7, zgc:112160, smpd5, casp7, ctsll, mmp28, ctss1, ctsd, notum2, casp8l2, nudt3b, mbtps1, tmprss13b, zgc:154142, zgc:92137, si:dkeyp-50d11.2, psmc1b, mmp11b, ptrh2, npsn,furina, cel.2*
Monooxygenase activity	8	0.006	*loc793236, ch25h, cyp24a1, cyp11c1, cyp1b1, cyp4v8, cyp7a1, cyp8b1*
Serine-type peptidaseactivity	7	0.018	*zgc:100868, cfd, zgc:112160, tmprss13b, zgc:154142, zgc:92745, furina*
Heme binding	8	0.018	*cyp24a1, cyp11c1, cyp1b1, duox, cyp4v8, cyp7a1, cyp8b1, ido1*
Serine-typeendopeptidaseactivity	9	0.020	*zgc:100868, cfd, mbtps1, zgc:112160, tmprss13b, zgc:154142, loc100149563, zgc:92745, furina*
Ferric iron binding	3	0.024	*loc558816, zgc:194125, zgc:109934*
Transporter activity	9	0.038	*aqp8a.2, rbp2b, rbp1, slc15a1a, slc13a5b, aqp7, ttpal, fabp11b, slc15a1b*
Transcription factor activity, RNApolymerase II core promoter proximal region sequence-specific binding	3	0.038	*fosl1a, fosab, jdp2b*
Metalloendopeptidase activity	6	0.048	*lmln, mmp11b, mmp28, c6ast3, npsn, c6ast4*
Oxidoreductase activity, acting on paired donors, with incorporation or reduction of molecular oxygen	6	0.049	*cyp24a1, cyp11c1, cyp1b1, cyp4v8, cyp7a1, cyp8b1*

Table S4: Primer sequences and conditions used in RT-qPCR.

Gene	Sequence 5′→3′ (F: forward; R: reverse)	Cycles	Annealing temperature (°C)	Amplicon lenght (pb)
cyp1b1	F: ACAGCTCTCCAGTGGATCAT<br>R: GTCTGCGATGGTTGGAAGAC	40	60	114
ef1α	F: CTGGAGGCCAGCTCAAACAT<br>R: ATCAAGAAGAGTAGTACCGCTAGCATTAC	40	60	87
igfbp1b	F: GGCACAGGAGAGCATCAAGT<br>R: GGTGAACTTCTCTCCCAACG	40	60	130
junbb	F: AAAATGGAGCAGCCGTTTTA<br>R: CTCGGTCACGTTCAAGTTCA	40	60	117
ubl7b	F: TTCGTACGGCATCAAGTCTG<br>R: GCCTGAAGCATCCTGAACTC	40	60	123
wdr35	F: TGCCGGAAAACCATAAACTG<br>R: CTGCTTTGGGCTGGTTACAT	40	60	108
rbp1	F: CCATCACTTGGGATGGAGAC<br>R: ATCTGTTTGCCCACAACTCC	40	60	133
acta1b	F: ATCTGGCTGGTCGTGATCTT<br>R: TCCTTGATGTCACGCACAAT	40	60	106
cyp24a1	F: CAGCCGGACCCTGGACAAAGATAC<br>R: CCAGCGCTCGGGTCTGAAC	40	60	139
